# Antioxidant Defence Systems and Oxidative Stress in Poultry Biology: An Update

**DOI:** 10.3390/antiox8070235

**Published:** 2019-07-22

**Authors:** Peter F. Surai, Ivan I. Kochish, Vladimir I. Fisinin, Michael T. Kidd

**Affiliations:** 1 Department of Microbiology and Biochemistry, Faculty of Veterinary Medicine, Trakia University, 6000 Stara Zagora, Bulgaria; 2 Department of Hygiene and Poultry Sciences, Moscow State Academy of Veterinary Medicine and Biotechnology named after K. I. Skryabin, 109472 Moscow, Russia; 3 Department of Animal Nutrition, Faculty of Agricultural and Environmental Sciences, Szent Istvan University, H-2103 Gödöllo, Hungary; 4 All-Russian Institute of Poultry Husbandry, 141311 Sergiev Posad, Russia; 5 Center of Excellence for Poultry Science, University of Arkansas, Fayetteville, AR 72701, USA

**Keywords:** antioxidants, poultry, oxidative stress, Nrf2, vitagenes

## Abstract

Poultry in commercial settings are exposed to a range of stressors. A growing body of information clearly indicates that excess ROS/RNS production and oxidative stress are major detrimental consequences of the most common commercial stressors in poultry production. During evolution, antioxidant defence systems were developed in poultry to survive in an oxygenated atmosphere. They include a complex network of internally synthesised (e.g., antioxidant enzymes, (glutathione) GSH, (coenzyme Q) CoQ) and externally supplied (vitamin E, carotenoids, etc.) antioxidants. In fact, all antioxidants in the body work cooperatively as a team to maintain optimal redox balance in the cell/body. This balance is a key element in providing the necessary conditions for cell signalling, a vital process for regulation of the expression of various genes, stress adaptation and homeostasis maintenance in the body. Since ROS/RNS are considered to be important signalling molecules, their concentration is strictly regulated by the antioxidant defence network in conjunction with various transcription factors and vitagenes. In fact, activation of vitagenes via such transcription factors as Nrf2 leads to an additional synthesis of an array of protective molecules which can deal with increased ROS/RNS production. Therefore, it is a challenging task to develop a system of optimal antioxidant supplementation to help growing/productive birds maintain effective antioxidant defences and redox balance in the body. On the one hand, antioxidants, such as vitamin E, or minerals (e.g., Se, Mn, Cu and Zn) are a compulsory part of the commercial pre-mixes for poultry, and, in most cases, are adequate to meet the physiological requirements in these elements. On the other hand, due to the aforementioned commercially relevant stressors, there is a need for additional support for the antioxidant system in poultry. This new direction in improving antioxidant defences for poultry in stress conditions is related to an opportunity to activate a range of vitagenes (via Nrf2-related mechanisms: superoxide dismutase, SOD; heme oxygenase-1, HO-1; GSH and thioredoxin, or other mechanisms: Heat shock protein (HSP)/heat shock factor (HSP), sirtuins, etc.) to maximise internal AO protection and redox balance maintenance. Therefore, the development of vitagene-regulating nutritional supplements is on the agenda of many commercial companies worldwide.

## 1. Introduction

Commercial poultry production is associated with a variety of environmental, technological, nutritional and biological/internal stressors which are responsible for decreased productive and reproductive performance and compromised health [1,2]. A great body of recent information clearly indicates that very often overproduction of free radicals, compromised antioxidant defences and oxidative stress are the leading causes of the detrimental consequences of stress in poultry. During evolution, integrated antioxidant defence systems were developed in poultry. These protective systems control free radical (i.e., ROS and RNS) production and maintain redox (antioxidant/prooxidant) balance. Indeed, the redox balance in the cell/whole body is shown to be responsible for a regulation of an array of various physiological/biochemical processes, including cell signalling, gene expression and homeostasis maintenance [3,4]. In our previous publications [5,6,7], the concept of the antioxidant defence network in poultry was developed and validated. It was suggested that there are three major levels of antioxidant defence in the cell. The first level is built by three major antioxidant enzymes, namely, superoxide dismutase (SOD), glutathione peroxidase (GPx) and catalase, which are responsible for radical detoxification at the very beginning of the process of their formation. Since free iron and copper are major catalysers of the free radical formation, metal-binding proteins were also placed into the first level of antioxidant defence. Because of a great variety and substantial number of free radical formation in biological systems, the first level of defence cannot deal effectively with all of them and, therefore, the second level of the antioxidant defence includes mainly free radical scavenging antioxidants (vitamin E, ascorbic acid, glutathione (GSH), CoQ, uric acid, etc.), with vitamin E being the major biological antioxidant in the cell membranes. However, even the second level of the antioxidant defence is not potent enough to prevent damage to biological molecules and, therefore, the third level of the antioxidant defence deals with damaged molecule repair (methionine sulfoxide reductase (Msr); heat shock proteins (HSPs); DNA-repairing enzymes, etc.) or removal (e.g., phospholipases, proteosomes). In recent years, the signalling role of free radicals has received much attention [8,9] and the vital functions of various transcription factors [10,11] and vitagenes [12,13] have been described. It is the aim of this review to present an updated assessment of antioxidant defence systems in poultry.

## 2. Stressors in Poultry Production

From a physiological point of view, deviation from optimal internal and external conditions leads to stress. Under mild stress conditions, homeostasis can be re-established by coordinated action of the hypothalamic–pituitary–adrenal system, the autonomic nervous system and the immune system. Further, a complex cascade of regulatory mechanisms is involved in the stress response which leads to metabolic changes (an increased mobilization of energy and a shift in metabolism) causing diminished live performance in poultry [14]. It should be mentioned that, in modern commercial poultry production, based on balanced diets and well-controlled environmental housing, stress-related nutritional metabolic diseases (e.g., encephalomalacia, exudative diathesis, muscular dystrophy) are sparse [6,7,15]; however, stress-related decreases in productive and reproductive performance of poultry still cause substantial economic losses. Domestication and genetic selection for rapid growth, improved feed conversion and high egg production rates have made domestic birds, including broilers, layers and turkeys, particularly susceptible to oxidative stress [16]. In general, there are four major types of stress in the poultry industry: technological, environmental, nutritional and internal, which lead to detrimental changes at the molecular/cellular and physiological levels and, finally, decrease the productive and reproductive performance of commercial poultry [1,2,15] (Table 1). 

A growing body of evidence clearly indicates that oxidative stress is involved in most of commercially relevant stresses in poultry production (for review see [1,2,15,60,86]).

## 3. Antioxidant Defence Systems

**Figure 1:**
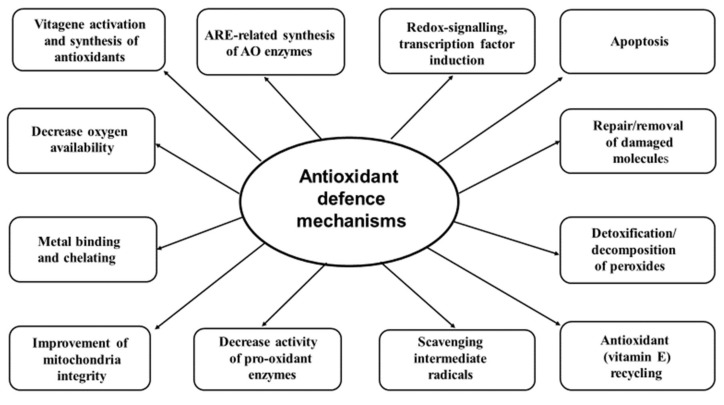
Antioxidant defence mechanisms (adapted from Reference [13]).

Living cells have to effectively balance the process of formation and inactivation/detoxification of ROS/RNS to maintain their levels low, but still above zero. It has been known for many years that cells can tolerate mild oxidative stress by additional synthesis of various antioxidants (GSH, thioredoxin (Trx), antioxidant enzymes, CoQ, etc.) and restoring redox antioxidant/prooxidant balance [87]. Since ROS in excess are damaging to many biological molecules, the antioxidant system network is responsible for prevention/decrease of the damages. However, these adaptive mechanisms in living organisms have limited ability. Once the ROS/RNS production exceeds the ability of the antioxidant defence system to neutralise them, oxidative stress occurs and important biological molecules, including polyunsaturated fatty acids (PAFAs), proteins and DNA can be damaged leading to detrimental consequences in terms of health, growth and development of poultry. Therefore, the antioxidant defence includes several options [13,15,88,89,90,91,92,93,94] (Figure 1):

An antioxidant strategy has several options. Firstly, there is an attempt to decrease free radical production by decreasing oxygen availability, reducing the activities of enzymes responsible for ROS/RNS production (e.g., NADPH oxidase, xanthine oxidase), keeping iron and copper bound to proteins and preventing their participation in new radical formation. Secondly, maintaining the integrity of mitochondria, the major source of free radicals in biological systems, is of paramount importance. Thirdly, scavenging free radicals (e.g., vitamin E, vitamin C, GSH, coenzyme Q) and detoxification/decomposition of the free radicals and non-radical toxic products (SOD, GPx, catalase, etc.) are important steps in the antioxidant defence strategy. Fourthly, the system of vitamin E recycling (ascorbic acid, thioredoxin reductase (TrxR), vitamins B1 and B2) which helps maintain vitamin E in an active form can increase its biological antioxidant potency. Fifthly, redox signalling, transcription factor (Nrf2) and vitagene activation and additional synthesis of protective molecules possessing antioxidant and detoxification activities are the main elements of the anti-stress strategy. Sixthly, enzymatic systems, responsible for damaged molecule repair (heat shock proteins, HSP; methionine sulfoxide reductase, Msr; DNA-repair enzymes; etc.) and removal (phospholipases, phospholipid hydroperoxide GPX (PH–GPx), proteasomes, etc.) play an important role in preventing the accumulation of damaged molecules and maintaining proteostasis. Finally, apoptosis, autophagy and other processes dealing with terminally damaged cells to remove them and prevent damages to be transferred to other cells/tissues are important elements of the antioxidant defence network.

Increasing evidence has demonstrated that all antioxidants in the body are working together as a “team” providing conditions for adaptive homeostasis. In this team, there are cooperative interactions when one member helps another one to work more efficiently. In fact, antioxidant defence systems are shown to be found in all cell compartments (e.g., mitochondria, nucleus, cytoplasm, etc.) and are expressed tissue-specifically, which includes internally synthesised antioxidants (AO enzymes, GSH, CoQ, uric acid, carnitine, taurine, etc.) and antioxidants supplied with the diet (vitamin E, carotenoids, synthetic antioxidants, carnitine, silymarin, etc.). Many antioxidant enzymes (e.g., SOD, GPx and some other selenoproteins, etc.) are stress-inducible and their expression and activity depend on stress intensity [15]. 

## 4. The Concept of Oxidative Stress

**Figure 2:**
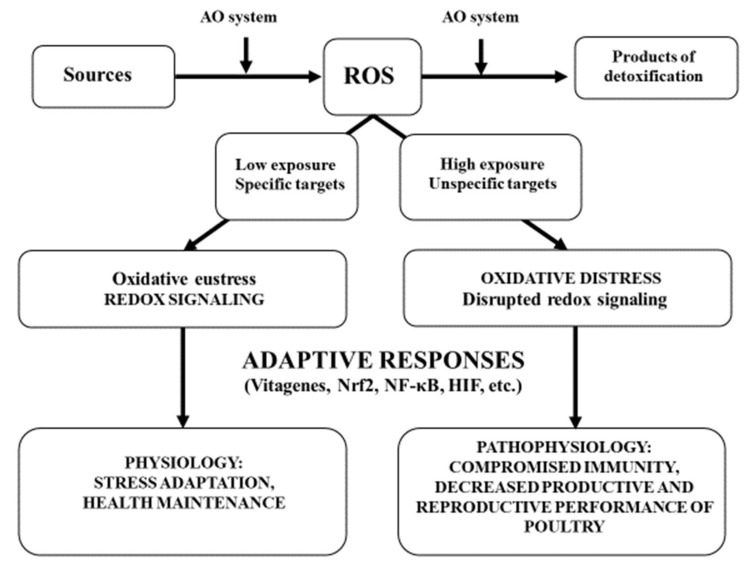
Oxidative stress and adaptive responses (adapted from Reference [99] with modifications).

The concept of oxidative stress as an imbalance between oxidants and antioxidants and oxidative stress responses was initially formulated in 1985 by Sies [95] and was later updated [96,97,98,99]. Terms introduced were “oxidative eustress”, referring to low-level physiological oxidative stress, and “oxidative distress”, referring to high-level oxidative stress [99]. The concept is graphically summarized in Figure 2. 

ROS are produced in physiological conditions as a by-product of energy production in mitochondria and as an important “weapon” in phagocytes. The antioxidant defence network is responsible for maintenance of low basic levels of ROS by scavenging and converting them into non-toxic products and other mechanisms (for details, see Figure 1). Low levels of ROS interact with specific targets and play important roles in redox signalling (i.e., oxidative eustress). Working closely with various transcription factors and vitagenes, these ROS are responsible for stress adaptation, homeostasis and health maintenance. High exposure to ROS due to the presence of compromised antioxidant defences or excessive ROS production as a result of high stress conditions affects unspecific targets (damaged PUFAs, proteins, DNA, etc.) and causes oxidative distress leading to disruption of redox signalling. This contributes to pathophysiology and leads to compromised immunity and decreased resistance to various diseases and the decreased productive and reproductive performance of poultry.

In response to the oxidative challenge, a stress response is activated to control potential overproduction of ROS/RNS and to provide optimal conditions for effective ROS/RNS signalling to support redox homeostasis. The NF-E2-related factor 2/Kelch-like ECH-associated protein 1 (Nrf2/Keap1) and nuclear factor kappa-light-chain-enhancer of activated B cells/inhibitory κB protein (NF-κB/IkB) systems were considered to be two major “master regulators” of the stress response. In particular, in stress conditions, both transcription factors are shown to be translocated to the nucleus, bound to appropriate DNA sites to provide protection, but in many cases with opposite effects. In general, the stress response is a quite complex process associated with various important biochemical and physiological pathways including the heat shock response, the unfolded protein response, the hypoxia induced response, and various repair mechanisms/programs. Furthermore, autophagy, mitophagy, apoptosis, necroptosis, ferroptosis, etc., are also deeply involved in homeostasis maintenance [99]. 

It is important to mention that the signalling roles of ROS have received a tremendous amount of attention [100] and it was suggested that they are produced in a strictly controlled/regulated fashion [101]. Growing evidence indicates that oxidation-reduction (redox)-based regulation of gene expression and adaptive homeostasis are fundamental regulatory mechanisms in cell biology. A great variety of protective systems against oxidative stress in poultry is suggested to be strictly regulated and, depending on the conditions, the stress response can be created over a period of minutes to hours, days to weeks, or months to years [102]. In this respect, signal transduction pathways such as the Nrf2-Keap1 system is one of the fastest responding systems to the changing environment which can upregulate the antioxidant defence network within minutes [102]. Mounting evidence has shown that detrimental alterations in redox signalling in stress conditions could lead to disease development [103] and losses in productive and reproductive poultry performances [15]. However, a low/basal level of oxidative stress is an essential part of cell adaptation and survival due to the creation of adaptive responses with improved adaptive ability to stressful challenges/conditions [104]. Recent findings indicate that in poultry, redox-signalling pathways use ROS as signalling molecules to activate expression of genes responsible for regulation of various physiological functions including growth, differentiation, proliferation and apoptosis, as well as to activate vitagenes and increase adaptability to stress [105]. Adaptive homeostasis was presented as a mechanism explaining how variations in stress exposure (type, duration, intensity/strength, etc.), including oxidative stress, are dealt with by a cooperative action of various protective mechanisms [102].

## 5. Vitagene Network

The term “vitagene” was first introduced by Rattan in 1998 to describe various maintenance and repair processes in the cell [106]. Hence, several important gene-coding proteins that regulate the complex network of the so-called longevity assurance processes were suggested to be called “*vitagenes*” [106]. Later, the vitagene concept was further developed in relation to the medical sciences by Calabrese and colleagues [107,108,109,110]. The major pro-survival mechanisms in the body/cells which are under vitagene network control are shown in Table 2. 

As can be seem from Table 2, the vitagene network operates on four levels. At the molecular level, it is related to antioxidant defence systems, including a DNA-repairing system, synthesis of stress proteins and proteasomal degradation of damaged proteins. Indeed, cellular proteostasis is a key element of homeostasis maintenance. Safety of genetic information transfer is also regulated at this level. At the cellular level, the aforementioned processes regulated at the vitagene level are related to cell proliferation and differentiation, and cell membrane integrity. Stability of intracellular milieu and macromolecular turnover are also connected to the vitagene network. At the tissue and organ level, the vitagene network is responsible for neutralization and removal of toxic chemicals, tissue regeneration, cell death and replacement. Therefore, the aforementioned events are vital elements of tissue/organ homeostasis maintenance. Finally, at the physiological level, the vitagene network is responsible for stress response, adaptation and thermoregulation. Furthermore, hormonal, immune and neuronal response to environmental/nutritional factors are also regulated by the vitagene response.

In accordance with Calabrese et al. [108,109,110], Surai and Fisinin [111,112] and Surai et al. [13] the term “vitagenes” includes a group of genes participating in cellular homeostasis preservation under stress conditions. Therefore, the vitagene family is suggested to include:

- Heat shock proteins (HSPs): HSP70 and heme oxigesnase-1 (HO-1);
- SOD;
- Thioredoxin system (Trx, Trx peroxidase (peroxiredoxins), sulfiredoxin and TrxR);
- Glutathione system (GSH, glutathione reductase (GR), glutaredoxin (Grx), GPx); and
- Sirtuins.

Data on the regulatory mechanisms of vitagene expression in poultry are quite limited. For example, HSP regulation in avian species has been recently reviewed [113] and can be summarised as follows. The heat shock response (HSR) is one of the main adaptive stress responses of the cell homeostasis restoration after proteotoxic stress, including heat shock, cold, oxidative stress, hypoxia, toxins, chemicals, pathogen, etc. [114,115,116]. In fact, cooperative interactions between the transcription factors and various homeostatic mechanisms are driving forces of effective adaptation to stressful conditions [117,118,119]. There is a growing body of evidence indicating the protective role of various natural antioxidants (e.g., vitamins E and C, carotenoids, flavonoids) in the prevention of lipid peroxidation and membrane integrity maintenance [15]. However, proteins can also be damaged in stress conditions and protein integrity preservation is the most important function of the stressed living cell/organism. Therefore, HSR in poultry/animals is based on the induction of HSPs and related elements, such as the ubiquitin–proteasome system [115]. In fact, HSPs are molecular chaperones facilitating protein folding and preventing protein aggregation in stress conditions [113]. Results of recent studies suggest that HSR is regulated mainly at the transcriptional level by four heat shock transcription factors (HSFs), including HSF1, HSF2, HSF3 and HSF4, which bind to heat shock regulatory elements of genes to upregulate HSPs’ expression [117]. Avian cells are known to express at least three HSFs (HSFs 1–3). In fact, three avian HSF genes corresponding to HSF3 as well as the avian homologs of mammalian HSF1 and HSF2 have been successfully cloned [120]. Furthermore, HSF1 was demonstrated to be rapidly activated by mild heat shock, while HSF3 was less responsive and responded only to severe heat shock. Therefore, HSF1 and HSF3 are quite different based on their activation kinetics and threshold induction temperature. Interestingly, HSF2 did not respond to heat stress and has been speculated to have other (developmental) functions [121]. In fact, HSF3 was considered to be a master regulator of the heat shock genes in avian cells [122]. Avian HSF1 and HSF3 were shown to be maintained in the cytoplasm in a cryptic monomer and dimer form, respectively, in physiological non-stressful conditions. Therefore, heat stress causes conformational change in chicken HSF3, associated with the formation of a trimer and its nuclear translocation [123].

It has been shown that avian cells lacking HSF1 and HSF3 have a complete loss of activation of heat shock genes under stress conditions [124]. Furthermore, HSF-deficient cells were also characterised by a dramatic reduction in HSP90α expression under normal growth conditions. There is a tissue specificity in HSF expression. For example, upon severe heat shock, HSF1 was shown to mediate transcriptional activity only in the brain. At the same time, HSF3 was found to be exclusively activated in blood cells as a result of heat treatment following induction of heat-shock genes [125]. It has been demonstrated that vertebrate HSF2 can be induced in the physiological range of temperature. In fact, HSF2 deficiency was found to reduce the threshold for chicken HSF3 activation, and HSF2-null cells became more susceptible to mild heat shock in comparison to wild-type cells. In addition, HSF2-deficient cells were characterised by the accumulation of ubiquitylated misfolded proteins [126]. In general, the vital roles of HSFs in the adaptation of poultry to commercially relevant stress conditions are proven. Recent genome-wide studies have provided information to appreciate the roles of HSF1 in reprogramming transcription not only in stress conditions but also in physiological conditions [127]. Detailed analysis of possible protective functions and regulation of HSP70 and HO-1 in avian species has been recently published [111,112,113] and can be summarised as follows.

### 5.1. HSP70

Among the HSPs, HSP70 is considered to be one of the most conserved and important protein families. In fact, HSP70 refers to a family of 70 kDa chaperone proteins participating in house-keeping functions. These ATP-dependent chaperones are key elements of the cellular protein surveillance network involved in a large variety of protein-folding processes [128]. It is well appreciated that under various stress conditions, adaptive synthesis of stress inducible HSP70 enhances the ability of stressed cells to maintain proteostasis by dealing with increased concentrations of unfolded or denatured proteins (for recent reviews, see References [129,130,131,132]). Organisation, nucleotide sequence and transcription of the chicken *HSP70* gene have been delineated by Morimoto et al. [133]. Indeed, the authors isolated a gene encoding a 70 kDa heat shock protein (HSP70) from a chicken genomic library and determined that the gene is quite conserved, since the order and spacing of the sequences were shown to share many features in common with the promoter for the human *HSP70* gene. Similar to mammals, the heat induced a time-dependent increase in HSP70 mRNA and protein in broiler chicken liver in vivo was observed [134]. The tissue- and age-dependent expression of HSP70 in broiler chicken embryos was affected not only by heat, but also by cold stress [135]. Therefore, increased HSP70 expression is believed to be an important adaptive mechanism to deal with oxidative stress-related changes in cell proteome under various stressful conditions [113]. Interestingly, HSP expression in the chicken gut is a vital mechanism of antioxidant protection [17] and there is a need for further research to understand molecular mechanisms of HSP70 regulation in avian species.

### 5.2. Heme Oxygenase-1

Heme Oxygenase-1, a 32 kDa protein, known as heat shock protein-32 (HSP32), is responsible for the degradation of haem with the formation of carbon monoxide (CO), biliverdin and free iron. Similar to HSP70, HO-1 is the stress-inducible isoform of the three HO isoforms described to date, providing a critical protective mechanism in avian systems responsible for adaptation to oxidative, inflammatory and cytotoxic stress [136,137]. In most tissues HO-1 is expressed at a relatively low house-keeping level and it can be induced by various oxidative stress-related insults including haem, ultraviolet light, heavy metals, cytokines, hydrogen peroxide, nitric oxide (NO) and glutathione depletion [138,139]. The vital role of HO-1 in adaptation to stress has been shown in HO-1-deficient animal models characterised by atypical pro-inflammatory immune response with increased apoptosis [113]. It seems likely that HO-1 synthesis is under strict hormonal control. Until now, research data on HO-1 expression and its protective actions in poultry production were quite limited. In fact, in the early 1990s, HO-1 was purified from chicken liver microsomes [140]. The apparent V_max_ of purified heme oxygenase, assayed under optimal conditions, was 580 U/mg protein, with a molecular weight of 33,000 Da [140]. It was shown that, similar to mammals, bird HO-1 induction in stress conditions was associated with various signalling pathways. Interestingly, increased HO-1 expression in chicken embryos between internal (day 19) and external pipping (day 20; [141]) is believed to be an important adaptive mechanism responsible for increased protection of tissues during this critical and stressful period of the ontogenesis [142]. Heme Oxygenase-1 is described in avian species; however, its response to different stressors in domesticated and wild birds are still poorly characterised [113] and needs further investigation.

Therefore, our critical analysis of recent data indicates that HSP70 and HO-1 expression in avian species effectively responds to commercially relevant stressors including heat stress, heavy metal stress, Se deficiency, chicken transportation and increased stocking density. It is proven that HSP expression can be effectively regulated by nutritional means, including vitamins E, C and D, carnitine, betaine [13,113] and some phytochemicals such as silymarin [91]. Indeed, these vitagenes (HSP70 and HO-1) are important elements responsible for adaptation of poultry to various stressors by maintaining cell proteostasis

### 5.3. SOD

SOD, as important vitagene, is the main driving force in cell/body adaptation to various commercially relevant stress conditions [93,111,112]. Since the superoxide radical is the main free radical produced in physiological conditions in the cell [143], SOD is believed to be the key element of the first level of antioxidant defence in the cell [15]. Recently, the protective roles of SOD in avian biology have been reviewed [93] and the main conclusions can be summarised as follows. SOD was discovered in 1969 by McCord and Fridovich [144] and this discovery opened a new era in free radical research. There are three isoforms of SOD in mammals, namely, cytosolic Cu, Zn-SOD, mitochondrial Mn-SOD, and extracellular SOD (EC-SOD; [15,93]). It is proven that additional synthesis of SOD under stress conditions is an adaptive mechanism to decrease ROS formation, prevent oxidative stress and maintain adaptive homeostasis [145]. However, if the stress is too high, SOD activity is usually decreased following apoptosis activation. Chicken SOD was first described and purified in the early 1970s. In fact, similar to mammals, chicken liver has two types of SOD, including mitochondrial (Mn-SOD) and cytosolic (Cu, Zn-SOD) enzymes [146]. The cytosolic SOD was shown to have an apparent molecular weight of 30,600 Da and to contain copper and zinc, being similar to the other eukaryotic Cu, Zn-SOD, while chicken mitochondrial SOD was found to have a molecular weight of 80,000 Da. SOD activity in avian species is tissue specific and was shown to depend on many different factors such as genetics, nutrition and various stress-related factors, including heat, heavy metals, mycotoxins and other toxicants [93]. In particular, SOD was found to provide an effective protection against lipid peroxidation in chicken embryonic tissues [147] and in semen [148]. There are complex interactions inside the antioxidant network of the cell/body to maintain homeostasis under stress conditions. Nutritional means of SOD upregulation in poultry production and physiological and commercial consequences of such upregulation await further investigation. For example, in the medical sciences, manipulation of SOD expression and SOD mimics are used as an important tool in disease prevention and treatment [93].

### 5.4. Sirtuins

Sirtuins (SIRTs) are a highly conserved family of NAD+-dependent enzymes possessing deacetylases, deacylase, mono-ADP-ribosyltransferase and other activities [149]. The role of sirtuins as an important part of the vitagene family in avian species has been recently reviewed [111,112] and can be summarized as follows. There are seven members of the sirtuin family, SIRT1–SIRT7, which are located in different subcellular compartments. These enzymes are ubiquitously distributed from eubacteria to mammals [150]. In particular, SIRTs have been associated with various cellular and metabolic processes regulating cell plasticity mechanisms of adaptation to various stresses [151]. Sirtuins are involved in regulation of redox balance in the cell by affecting specific transcription factors [152]. In fact, SIRTs orchestrate cellular stress response and maintain genome integrity and protein stability [153]. Indeed, a number of biological processes, including cell growth and differentiations, apoptosis, chromatin condensation, energy transduction and glucose homeostasis are regulated via SIRTs expression [149]. Furthermore, DNA repair and apoptosis [154], muscle and fat differentiation, neurogenesis, mitochondrial biogenesis, glucose and insulin homeostasis, hormone secretion, cell stress responses and circadian rhythms are proven to be regulated by SIRTs [155,156]. In fact, main cytosolic and nuclear targets of SIRT1 are shown to include histones, p53, DNA damage proteins, FOXO1, -3 and -4, HSF1, PPARγ, PPARα, UCP2, NF-κB and HIF1α [157]. Therefore, sirtuins are deeply involved in various stress-related pathways within the complex signalling network responsible for regulation of stress response and restoration of adaptive homeostasis under stress conditions [151,152]. Research assessing SIRT expression in poultry is sparse, but accumulating evidence indicates that SIRTs are highly conserved among organisms [158]. Indeed, *SIRT1* activation in goose hepatocytes in vitro was found to decrease fatty acids synthesis and cell proliferation and increased fatty acids oxidation. Interestingly, SIRT1 inhibition had an opposite effect [159]. Similar to mammals, stress can increase SIRT expression in birds, e.g., there was an upregulation of SIRT1 in the chicken hypothalamus, liver and muscle in response to 48 h fasting [160]. On the other hand, heat shock (HS) was shown to downregulate SIRT1 in the chicken liver, while dietary supplementation of epigallocatechin gallate ameliorated the detrimental effects of HS on SIRT1 expression [161]. The expression and regulation of sirtuin family members in chicken liver have been characterised [162]. In particular, it was shown that chicken SIRTs share the same conserved functional SIR2 domains. The chicken sirtuins are located in various cellular compartments, including the nucleus (cSIRT3 and cSIRT5), cytoplasm (cSIRT2 and cSIRT4), and in both the cytoplasm and nucleus (cSIRT1, cSIRT6 and cSIRT7). All sirtuins except cSIRT7 were characterised by a deacetylase activity. It was predicted that chicken sirtuins play roles in central intermediary metabolism (cSIRT1, cSIRT2, cSIRT5 and cSIRT6) and in amino acid biosynthesis (cSIRT3). Although cSIRT7 does not possess enzymatic properties, cSIRT4 has been suggested to participate in transcription regulation, with potential regulatory functions. In 30 week old laying hens, SIRTs were found to be expressed in the heart, liver, pectoralis, kidney, spleen, abdominal fat, duodenum, glandular stomach, pancreas and lungs. An age-related regulation of gene expression (with increasing with sexual maturity) of *cSIRT1*, *cSIRT2*, *cSIRT4*, *cSIRT6* and *cSIRT7* was observed in the chicken liver [162]. Recently, 24 target genes of SIRT1 in chicken embryonic liver were identified. These genes are responsible for the activation or inhibition of lipolysis and gluconeogenesis in embryos [163]. Because of their roles in cellular stress responses, sirtuins would be expected to be important players in adaptive responses of poultry to stress and this topic awaits further investigation. It seems likely that major vitagenes in poultry, which encode elements of thioredoxin and glutathione systems, are regulated via a Nrf2 system, and they will be characterised in the next section.

The products of the aforementioned vitagenes are believed to be involved in the detection and creation of a protective response to diverse forms of stress and cell injuries. The molecular mechanisms of the vitagene network operation in various stress conditions have been recently reviewed [110,164], and the biochemical and physiological consequences of the upregulation of the vitagene network in cells and the organism as whole is an important direction of current research [12,165,166]. In accordance with the above reports, cellular stress response could be modified by vitagene activation leading to additional synthesis of various protective antioxidant molecules helping effective stress adaptation. The vitagene concept already found its acceptance in the medical sciences in relation to neurodegenerative disorders [107], neuroprotection [109,167], autism [168], dermatology [169], osteoporosis and Alzheimer pathology [165,166,170,171,172], schizophrenia [12], free radical-related diseases [173,174], aging and longevity [108,110,175,176].

A number of findings clearly indicate that the vitagene concept can also be valid in poultry [177,178,179]. Indeed, the vitagene concept was further developed and validated in relation to poultry production [13,89,90,91,92,112,113,179]. Accumulating experimental evidence indicates that there is a great opportunity to nutritionally modulate the vitagene network using various nutritional supplements, including phytochemicals [176], carnitine, [90,180], betaine, taurine and vitamins A, E and D [13,181]. In fact, activation of the vitagene network by nutritional means is considered a new fundamental approach for improving animal/poultry resistance to various stresses [13,111].

## 6. Transcription Factor Nrf2

It is well appreciated that antioxidant defence systems are under strict regulation by a number of transcription factors [182,183,184,185]. In fact, oxidative stress is believed to activate a range of transcription factors/pathways including Nrf2, NF-κB, AP1, TP53, HIF-1α, HSF1, PPAR-γ, MAPK, FoxO, NOTCH, CREB, SCREB1, SP, β-catenin/Wnt, etc. [186,187,188,189]. Activation of these transcription factors is shown to be associated with the expression of over 500 different genes [186]. They modulate the antioxidant defence network by effecting ROS-generating and antioxidant enzymes, mentioned above, which are critically important for animal adaptation to various stresses. In recent years great attention has been paid to a basic leucine zipper transcription factor, Nuclear factor-erythroid-2- (NF-E2-) and related factor 2 (Nrf2) which will be considered briefly in this section. It is well established that Nrf2 is the redox-sensitive master regulator of oxidative stress-related signalling responsible for the adaptive stress responses [190]. Clearly, Nrf2 has a vital role in adaptation to oxidative stress via induction of the expression of various protective molecules [191,192,193,194].

**Figure 3:**
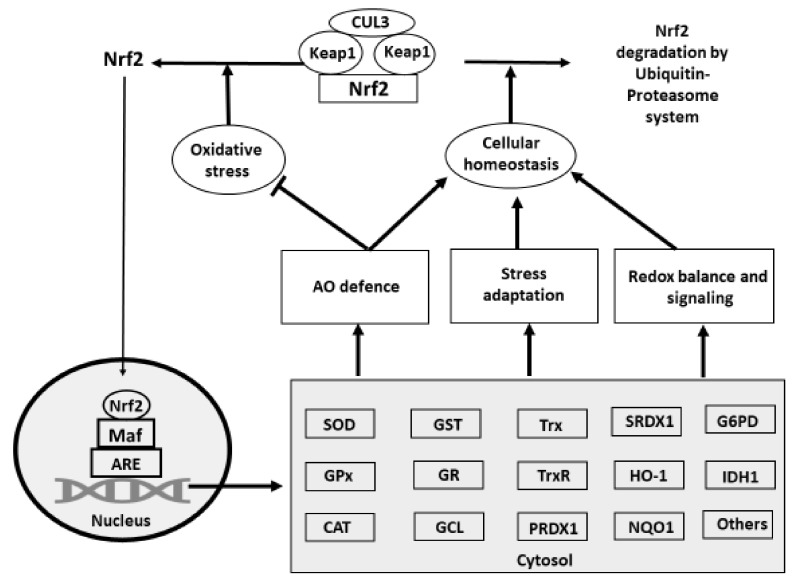
Participation of Nrf2 in the AO defence network. In cells under physiological homeostatic conditions, cytosolic transcription factor Nrf2 is kept at low levels being bound to Keap1 by the ubiquitin ligase complex Cullin (Cul)3-RING-box protein (Rbx)1 (Cul3). This complex ubiquitinates Nrf2, triggering its constant proteasomal degradation. Under oxidative stress, ROS modify/oxidise SH-groups within Keap1 leading to conformational changes inducing the Nrf2 release from Keap1. This prevents Nrf2 proteasomal degradation and Nrf2 translocates to the nucleus. In the nucleus, Nfr2 binds to the ARE and initiates the transcription of an array of direct or indirect antioxidant enzymes including SOD, GPx, CAT, GST, GR, GCL, Trx, TrxR, PRDX1, SRDX1, HO-1, NQO1. G6PD, IDH2, etc. These enzymes contribute to the improvement of the antioxidant defence network and reduce the cellular oxidative stress. The Nrf2 induced synthesis of AO enzymes also participates in regulation of stress adaptation and redox signalling. The restoration of cellular homeostasis leads to Nrf2–Keap-1 complex formation and activation of Nrf2 degradation by ubiquitin–proteasome system and decreases the Nrf2 mediated synthesis of AO enzymes.

The existing evidence suggests that under normal physiological conditions, Nrf2 represents a 605 amino acid transcription factor located in the cytoplasm. It is shown to consist of seven functional domains (Neh1-7) responsible for the regulation of its stability or/and transcriptional activity. In normal physiological conditions, Nrf2 exists as an inactive complex with the negative regulator called Kelch-like-ECH-associated protein 1 (Keap1): a 624 amino acid, cysteine-rich, homodimeric zinc-finger protein [195]. In fact, Keap1 is responsible for forwarding Nrf2 to a Cul3-based E3 ligase with the following rapid 26S proteasome degradation with a half-life under physiological conditions of only ∼20 min [196,197]. Importantly, more than 27 cysteine residues possessing different reactivity and having various functional impact on Nrf2 were identified in Keap1. In fact, Cys151, located within the BTB homodimerization domain, was shown to be responsible for Nfr2 activation. Furthermore, other cysteines (Cys273, 288 and 297) located in the intervening region, were indicated to inhibit Nrf2 activity by promoting its interaction with Keap1. Furthermore, seven redox-sensitive cysteines (Cys119, 235, 311, 316, 414 and 516) have also been identified in Nrf2 and their oxidative modification could also affect its activity [198]. Therefore, Keap1 serves as an important cellular redox sensor participating in redox balance regulation via its interaction with Nrf2 [195]. It is generally believed that oxidative or electrophilic stress, causing increased ROS production, can modify/oxidize critical cysteine thiols of Keap1 leading to the Keap1-Nrf2 complex dissociation and prevention of Nrf2 degradation in proteasome. Therefore, Nrf2 translocates to the nucleus, heterodimerizes with one of the small Maf (musculoaponeurotic fibrosarcoma oncogene homolog) proteins and binds to antioxidant/electrophile-response elements (ARE) in the upstream promoter region of genes encoding various antioxidant molecules, see Figure 3 in Reference [195].

There is a range of other mechanisms promoting Nrf2 activation, including its phosphorylation associated with a Nfr2–Keap1 dissociation and Nrf2 translocation to nucleus [199]. In particular, at least four different mechanisms are described which could lead to dissociation of Keap1 from Nrf2: oxidation of cysteine residues (lower molecular weight reactive oxygen species); covalent modification of cysteine residues (electrophiles); phosphorylation of Nrf2 at Ser40 by protein kinase C and PERK; and protein–protein interaction between p62 and Keap1 [200]. Indeed, Nrf2 activation is responsible for the regulation of multiple pathways via direct inactivation of oxidants, increasing levels of GSH, Trx and NADPH synthesis, enhancing toxin export via the multidrug response transporters, inhibiting cytokine-mediated inflammation, enhancing recognition, repair and removal of damaged proteins, increasing chaperones and regulating posttranslational protein modifications (Table 3) [201,202].

Indeed, Nrf2 initiates synthesis of enzymes of the first line of the antioxidant defence, namely, SOD, GPx and catalase. They deal with free radicals at the site of their production. Since the superoxide radical is the main radical in the biological system, its effective removal is a primary task of the first level of the antioxidant defence [93]. However, as a result of SOD action, H_2_O_2_ is produced, which is mainly dealt with by GPx, which has been recently characterised in relation to poultry [205,206]. Glutathione (GSH) is the most abundant non-protein thiol in avian and mammalian cells controlling redox balance and signalling, regulating transcription factors and gene expression and many other important cellular pathways/processes including epigenetic mechanisms [207].

It is well known that GSH can be synthesized in poultry from three amino acids (i.e., L-glutamate, L-cysteine and glycine) with glutamate cysteine ligase (GCL) being the rate-limiting enzyme in GSH biogenesis [208]. Therefore, Nrf2-regulated synthesis of GCL is of great importance for the antioxidant defence network efficacy. Indeed, GSH is exclusively synthesized in cytosol and compartmentalized in different organelles, including nuclei, endoplasmic reticulum (ER) and mitochondria. In particular, nuclear GSH is found exclusively in the reduced form and participating in preserving proteins involved in DNA repair and gene transcription. Furthermore, mitochondrial GSH preserves the mitochondrial integrity by controlling mitochondrial ROS generation and apoptotic signalling [209]. Therefore, cellular GSH is a key regulator of different biological processes, including synthesis of DNA and proteins, affecting cell growth and proliferation, apoptosis, immunity, amino acid transport, xenobiotic and endogenous oxidant metabolism/detoxification, redox-sensitive signal transduction, etc. [210,211]. On the one hand, the GSH thiolic group can directly react with and detoxify a range of ROS, including H_2_O_2_, superoxide anion, hydroxyl radicals, alkoxyl radicals and hydroperoxides [209], on the other hand, there is a range of proteins with GSH-dependent hydroperoxidase activity, including GPx, peroxiredoxins (Prx)-isoforms, some Grx and many GST [212]. Importantly, in stress conditions GSH plays a vital role as a redox buffer (GSH/GSSG; [212]) controlling the redox status of the living cells, responsible for prevention of the loss of protein thiols and providing optimal redox milieu for signalling [213], either by protein glutathionylation (direct modification of protein cysteine residues by the addition of GSH) or via scavenging hydrogen peroxide [214]. Indeed, the ratio of GSH/GSSG is the main indictor of the cellular redox potential and reflects redox balance. Under oxidative stress, a decreased redox potential (GSH/GSSG ratio) causes protein S-glutathionylation: a mixed disulphide formation between reactive thiols and GSH altering the physiological functions of affected proteins. In fact, abnormal protein S-glutathionylation is related to diverse cellular detrimental changes, including protein aggregation, protein degradation, apoptosis and mitochondrial dysfunction [215].

A thiol redox system consisting of the glutathione system (glutathione/glutathione reductase/glutaredoxin/glutathione peroxidase) and the thioredoxin system (thioredoxin/thioredoxin peroxidase (peroxiredoxins)/sulfiredoxin/thioredoxin reductase [216] are believed to be the major players in redox status regulation [99,217,218,219]. Further, the thioredoxin system is an important thiol/disulphide redox controller ensuring the redox homeostasis [220]. Numerous studies have clearly demonstrated that the thioredoxin system is involved in the redox regulation of the expression of genes regulating various cellular functions, including synthesis of deoxyribonucleotides (DNA synthesis and repair), protein biosynthesis, hormone and cytokine action, apoptosis, etc. [221]. Therefore, Nrf2-regulated synthesis of major members of thioredoxin system is a key element of the anti-stress strategy in the cell/body [111].

Chicken Trx is a protein of 105 amino acids with a molecular weight of 11,700 [222]. The sequence of the chicken Trx is shown to be very similar to the sequences of other thioredoxins. In particular, comparison of the chicken Trx protein sequence with those from bacteria and plants showed structural features that appear to be essential for activity. Indeed, chicken *Trx2* is proved to be an essential gene and Trx2-deficient cells undergo apoptosis upon repression of the Trx2 transgene and accumulation of intracellular ROS [223,224]. Interestingly, increased Trx expression in chicken ovarian follicles was found to be associated with high rates of egg production [225]. Trx was shown to be expressed in chicken jejunum [226] and was indicated to be an important protein of the chicken seminal plasma [227]. Furthermore, chicken mitochondrial Trx2 was discovered to have disulphide reductase activity in a concentration-dependent manner providing protective effects on LPS-induced oxidative stress in chicken hepatocytes [228]. Trx silencing in chicken cardiomyocytes was shown to cause endoplasmic reticulum oxidative stress by modulating Ca^2+^ channel-related pathway genes [229]. It is well known that TrxR is a Se-dependent enzyme, but data on its activity in avian species are quite limited [15]. For example, Smith et al. [230] compared TrxR activity in mammals and chickens and showed that chickens have extremely low TrxR activities, probably reflecting low TrxR protein expression or reflecting differences between mammalian and chicken TrxR. Furthermore, TrxR activity was detected in a range of chicken tissues, including liver, lung, heart, kidney, brain, breast muscle, bursa, thymus, spleen, RBC and plasma [231]. TrxR activity was detected in association with the cytosolic, nuclear pellet and mitochondrial fractions. Selenium dietary supplementation (0.4 mg/kg diet) increased TrxR activity in duodenal mucosa, liver and in the kidney in chickens [232], while Se deficiency was shown to decrease expression/activity of TrxR in chicken thyroids [233], pancreas [234], adipose tissue [235], kidney [236] and duodenum mucosa [237]. High dietary fluorine was found to decrease TrxR activity in chicken serum and tissues [238]. Similarly, dietary lead was associated with a decreased TrxR activity in chicken brain [239]. Heat stress was shown to compromise the mitochondrial thioredoxin system including Trx2, TrxR2 and Prx3 in growing chickens and dietary curcumin can mitigate this detrimental effect [240].

At least four different classes of Prx protein have been shown to be evolutionary conserved in chickens [241]. In fact, chicken Prx proteins possess antioxidant activity; however, Prx expression in chickens is not tissue specific, showing their essential role as a housekeeping gene in all tissues to protect against oxidative damage [241]. Prx1 was found to be expressed in chicken macrophages [242], chicken embryonic kidney [243] and chicken jejunum [226]. Furthermore, chicken Prx6 was shown to be expressed in chicken liver [244] and chicken gut [245]. Acute heat stress was shown to upregulate Prx1 and Prx3 in the small yellow follicles of layer-type chickens [246]. Therefore, in poultry Trxs, Prxs and TrxRs can function as signal transduction proteins regulating stress-induced signalling cascades. They are important antioxidants participating in cellular/organismal adaptation to stress and their upregulation is considered to be an important approach to improve stress resistance of poultry.

Detoxification enzymes (HO-1, NQO1, and GST), synthesized under Nrf2 supervision, are a great help for antioxidant defence, as they are responsible for the prevention of the participation of various xenobiotics and heme in free radical production [127,247,248,249]. Importantly, Nrf2 was shown to restrict/prevent iron- or heme-mediated oxidative stress by affecting the expression of the *FPN1*, ferritin and *HO-1* genes [250]. Regulation of the aforementioned antioxidants, NADPH-synthesizing enzymes, as well as others stress-response proteins are clearly shown to provide protection against oxidative and inflammatory damages [197,203,204,251]. Accumulating evidence clearly shows that Nrf2 can also regulate many important biological processes, including cell proliferation and differentiation, inflammation, autophagy, apoptosis, mitochondrial function or biogenesis as well as several metabolic pathways involved in iron/heme, glucose, glutamine, lipid, NADPH and pentose phosphate metabolism (for review and references, see Reference [198]).

Generally speaking, Nrf2-mediated regulation of the antioxidant defence network and redox balance are key elements of adaptive homeostasis, with Nrf2 being involved in the regulation of the expression of about 250 genes [204]. In accordance with the earlier study, Nrf2 was reported to directly or indirectly alter the expression of approximately 15,000 genes, and the number of directly inducible genes has been estimated at 654. Interestingly, the inducible targets of Nrf2 are primarily categorized as antioxidant-related genes [201]. Therefore, a well-controlled adaptive change in gene expression as a response to stress via Nrf2 and the ARE is considered to be a key protective mechanism of homeostasis maintenance [203,204].

It has become increasingly apparent that stress stimulus activating Nrf2 includes redox disturbances, endoplasmic reticulum stress, autophagy impairment, inflammation, growth factor stimulation and nutrient/energy fluxes, etc. [195,252]. It is proven that beyond antioxidant defences Nrf2 also upregulates genes responsible for the synthesis of protective proteins directing the repair and degradation of damaged macromolecules during stress. Furthermore, it also modulates intermediary metabolism by direct metabolic reprogramming [251]. In addition, Nrf2 is involved in protein quality control by induction of proteasome subunits associated with a decrease in unfolded proteins and restoring physiological protein turnover [195].

It has been suggested that the Keap1/Nrf2 system predominantly senses and deals with low intensity oxidative stress, while intermediate oxidative stress more likely induces NF-κB and AP-1 pathways [189]. At low or intermediate intensity oxidative stress, additional adaptive mechanisms leading to enhanced antioxidant potential are related to MAP-kinases and other kinases (e.g., protein kinase C and phosphatidylinositol-3-kinase) participating in signal sensing and orchestrating cellular response with enhanced antioxidant potential [251]. Furthermore, emerging evidence clearly indicates that Nrf2 can interact with other transcription factors, including heat shock factor (Hsf1; [253]) to create additional options for AO system regulation. The Nrf2 stress pathway communicates with mitochondria, the main source of free radicals in biological system, to control cellular homeostasis during oxidative stress [254]. Based on the chemical structures, at least 10 classes of Nrf2 activators can be established, including diphenols, Michael reaction acceptors, isothiocyanates, thiocarbamates, trivalent arsenicals, 1,2-dithiole-3-thiones, hydroperoxides, vicinal dimercaptans, heavy metals and polyenes [255]. There is also a range of endogenously produced signalling mediators (e.g., H_2_O_2_, NO, fumarate, products of lipid peroxidation) effectively activating Nrf2 [255]. Currently, a number of natural and synthetic Nrf2-activating compounds have been described, and there is a growing body of evidence demonstrating the beneficial effects of Nrf2 activation in various stress conditions [204].

## 7. Protective Effects of Nrf2 in Poultry

A regulatory role for Nrf2 in stress adaptation in poultry has received limited attention and the main published studies related to Nrf2 expression in poultry tissues in various stress conditions appeared only in the last 10 years. They can be summarised as follows:

### 7.1. Heat Stress

The impact of heat stress on poultry performance is well documented, and the following text deals with the literature that delineates heat stress on poultry antioxidant capacity. Rearing five-week-old female Japanese quail at 34 °C for 8 h/d (HS) for 12 weeks was associated with a decreased production performance (a reduction in feed intake (FI) by 9.7% and egg production by 14.4%) and oxidative stress as evidenced by an increased hepatic MDA level by 84.8%, and decreased hepatic AO enzyme activities (SOD, CAT and GPx by 25.8%, 52.3% and 45.5%, respectively) [256]. At the same time, decreased hepatic Nrf2 expression was observed. In contrast, inclusion of epigallocatechin-3-gallate at 200 or 400 mg/kg in the quail diet restored the altered expression of Nrf2 by HS and ameliorated disturbances in AO enzyme activities [256]. In another experiment, conducted in the same department, one-day-old male broiler chicks (Ross 308) were randomly distributed to one of 2 × 3 factorially arranged treatments: two housing temperatures (22 °C for 24 h/d; thermoneutral (TN) or 34 °C for 8 h/d, HS) and three dietary lycopene levels (0, 200 or 400 mg/kg) and birds were reared to 42 days. Similar to the previous study, heat stress was associated with reductions in FI and weight gain by 12.2% and 20.7% and negatively affected FCR. In an HS group, oxidative stress was evidenced by decreased serum AO enzyme activities (SOD and GPx) and increased MDA. Furthermore, HS increased (by 150%) muscle Keap1 expression and decreased by 40% muscle Nrf2 expression. The authors showed that increasing dietary lycopene levels alleviated the detrimental changes in the Nrf2 system due to the HS and had a protective effect on chicken performance [257]. The detrimental effects of HS on Nrf2 expression in chicken muscles was partly alleviated by dietary Cr supplementation [258]. The protective effects of other phytochemicals against Nrf2-related AO enzyme changes due to the presence of HS were also reported. For example, curcumin supplementation (50–200 mg/kg) was shown to improve AO defences of HS-exposed broilers, as evidenced by increasing the GSH content and GSH-related enzyme activities and inducing the expression of Nrf2 and Nrf2-mediated phase II detoxifying enzyme genes [259]. Interestingly, birds in the HS group showed increased MDA, protein carbonyl (PC), 8-hydroxydeoxyguanosine (8-OHdG) and some apoptosis markers, including caspase-3 and caspase-9 mRNA levels and activity. In addition, the AO system was compromised (decreased GSH, Nrf2, GPx, MnSOD, HO-1, GR levels and compromised total antioxidant capacity (T-AOC), total SOD, Mn-SOD and catalase activities), reflecting HS-induced oxidative stress [260]. The authors clearly showed that resveratrol (400 mg/kg) was able to ameliorate HS-induced spleen dysplasia in broilers through the activation of the Nrf2 signalling pathway and decreasing apoptosis in the spleen. Similarly, dietary taurine (5 g/kg) was shown to significantly decrease the levels of ROS and MDA and increase the messenger RNA expressions of Nrf2, NAD(P)H quinone dehydrogenase 1 and HO-1 in breast muscles of HS-exposed birds [261]. It seems likely that the effect of HS on Nrf2 expression is condition dependent. For example, in another study, where 14 day old chickens were exposed to 35 °C for 12 days, Nrf2 was only slightly downregulated at day 1 post-HS compared to controls [262]. However, there was no difference in mRNA expression of Nrf2 at 12 days post-HS. Furthermore, in comparison to control, mRNA levels of SOD1 and CAT were downregulated at day 1 post-HS but were upregulated at day 12 post-HS [262]. Similarly, cold stress was found to increase Nrf2 protein expression in chicken liver [35].

### 7.2. Mycotoxins

In ovo exposure to aflatoxin B1 (AFB1) was shown to upregulate Nrf2 expression in both domesticated and wild turkey embryos [263]. In primary broiler hepatocytes, AFB1 caused increased mitochondrial ROS production, decreased mitochondrial membrane potential and induced apoptosis. This was associated with upregulated mRNA expression of Nrf2, but downregulated mRNA expressions of NAD(P)H: quinine oxidoreductase 1, SOD and HO-1 [264]. Similar changes in Nrf2 expression due to the exposure to AFB1 were also observed in broiler cardiomyocytes [265]. Interestingly, an opposite effect of AFB1 on Nrf2 expression was observed in vivo. For example, AFB1 (5 mg/kg for 28 days) induced liver injury in broilers and significantly downregulated Nrf2 and its downstream genes’ mRNA expression level. Moreover, the Nrf2 protein expression level was also markedly reduced in the AFB1-fed group [266]. Similarly, in broiler chick liver, dietary AFB1 (5 mg/kg for 28 days) significantly inhibited autophagy, induced inflammation and significantly reduced Nrf2 and HO-1 mRNA and protein levels [267,268]. However, curcumin dietary supplementation (150–450 mg/kg) was shown to significantly ameliorate AFB1-induced decreases in Nrf2 and HO-1 mRNA and protein expression levels. In contrast, in three-week-old broilers, ochratoxin A (OTA) exposure (1126 µg/kg feed) was associated with overexpression of the Nrf2 gene in liver and kidney as compared to control chickens [50]. Interestingly, in mice T-2 toxin exposure was shown to downregulate Nrf2 and its downstream target genes, including *NQO1* and *HO-1* [269]. In contrast, deoxynivalenol (DON) treatment in mice during pregnancy was found to cause ROS accumulation in the placenta leading to embryotoxicity. At the same time, the Nrf2/HO-1 pathway was upregulated in an attempt to protect placenta cells from oxidative damage [270]. Therefore, it seems likely that oxidative stress imposed by various mycotoxins is associated with Nrf2 up- or downregulation depending on the level of stress.

### 7.3. Heavy Metals

In an in vitro study, Se treatment was shown to increase the mRNA levels of Nrf2 in Cd-treated chicken hepatocytes [271]. However, in an in vivo study with chickens, it was shown that a high level of Se dietary supplementation (2 mg/kg as sodium selenite) was associated with significantly decreased accumulation of Nrf2 in the spleen nucleus compared with the corresponding control group, while Cd exposure increased nuclear Nrf2 accumulation [272]. In layer oviduct magnum epithelial cells in culture, vanadium downregulated Nrf2, NQO1 and HO-1 mRNA expression [273]. When laying hens were fed with four experimental diets containing graded levels of mercury at 0.280, 3.325, 9.415 and 27.240 mg/kg, respectively, an inhibitory effect of Hg on the Nrf2 protein level in ovary tissue was observed, while expression of Keap1 protein increased [274]. Similar inhibitory effects of Hg on Nrf2 expression was observed in the layer liver and kidney [275]. Therefore, the authors concluded that Hg causes damage to liver and kidney as a result of induced hepatic and renal oxidative stress due to the suppressing Nrf2–Keap1 signalling pathway in laying hens. In layer uterus gene expressions of Nrf2, and HO-1 were downregulated by vanadium treatment, while dietary epigallocatechin-3-gallate was able to upregulate the expression of the *Nrf2* and *HO-1* genes compared to the vanadium-exposed group [276]. Therefore, the aforementioned data clearly showed that heavy metals in a chicken diet caused the downregulation of the expression of Nrf2 leading to oxidative stress.

### 7.4. Lipopolysaccharide Challenge

In the liver of broiler chickens challenged with lipopolysaccharide (LPS), downregulated mRNA expressions of Nrf2, Cu/Zn-SOD, Mn-SOD, GPx1 and CAT were observed. However, dietary inclusion of oridonin, a compound extracted from medicinal herbs, was able to ameliorate the aforementioned effects of LPS on the antioxidant enzymes and Nrf2 expression [277]. Lipopolysaccharide treatment was shown to significantly increase the expressions of *Nrf2*, *Keap1*, *SOD1*, *SOD2*, *GPx* and *YAP1* genes in chick chorioallantoic membrane. Interestingly, addition of 400 or 800 μg/ml N-acetylcysteine suppressed the LPS-enhanced expressions of *Nrf2*, *GPx* and *Keap1* genes [278]. It seems likely that, similar to other stress conditions, the response of Nrf2 can be up- or downregulated by LPS.

### 7.5. Other Pro-Oxidants

Effects of corn-dried distillers’ grains with solubles (DDGS) on oxidative status in laying ducks was studied. It was shown that increasing corn DDGS (from 6% to 30%) linearly increased hepatic expression of Nrf2, HO-1 and GPX1, hepatic activity of GPx and the liver content of MDA [279]. Iron dietary supplementation at 700 or 1400 mg Fe/kg was shown to significantly increase *Nrf2* gene expression in jejunum of 21 day old Chinese fast growing Yellow broilers [280]. The expression levels of Nrf2 and HO-1 in goose granulosa cells treated with 3-nitropropionic acid were elevated 1.63 and 10.48 fold, respectively [281]. The mRNA level of Nrf2 in the liver and jejunum of broilers fed a diet containing soybean meal (SBM) heated at 100 °C for 8 h was significantly decreased in comparison to birds fed the control diet containing untreated SBM for 42 days [282]. The oxidative stress imposed by heat-treated SBM was associated with the decreased activities of SOD and GPx in liver and jejunum. Apoptotic, antioxidant, biochemical and histochemical alterations induced by boron administration in ostrich chicks’ kidneys were studied. For this purpose, the ostrich chicks were supplemented with boric acid (BA) (source of boron) in the drinking water at 0, 40, 80, 160, 320 and 640 mg/L [283]. As the boron concentration in the drinking water increased, the expression of *Nrf2* and *HO-1* genes in ostrich kidney were found to be upregulated reaching a maximum in the 80–160 mg/L boric acid groups. Further increases in boron supplementation were associated with the trend of downregulation of *Nrf2* and *HO-1* expression [283]. In conclusion, in most studies, mild nutritional stress was associated with increased *Nrf2* expression as an adaptive response. However, a long-term action of a stressor (heat-treated soybean meal) was associated with decreased *Nrf2* expression.

### 7.6. Other Stress Conditions

In a recent study with newly hatched chicks, *Nrf2* expression in the cerebrum of both the transport and simulation transport groups were significantly upregulated by transport stress [284]. However, the upregulation of *Nrf2* expression was not able to mitigate cerebrum oxidative stress in newly hatched chicks. Low-current and high-frequency electrical stunning was shown to create oxidative stress and increase lipid peroxidation. In such conditions, increased *Nrf2* gene expression in breast muscle of broilers due to the electrical stunning was not fully able to protect tissues against the aforementioned oxidative stress [285].

### 7.7. Phytochemicals

Recent data indicate that Nrf2 activation is one of the most important mechanisms of phytochemical protective effects in poultry and farm animals [91,286,287]. Dietary inclusion of *Antrodia cinnamomea* (a medical fungus) powder into the broiler diet at 0.1–0.4% for 35 days was shown to increase *Nrf2* expression in the liver [288]. Primary chicken intestinal epithelial cells treated with 100 nM equol showed increased abundance of Nrf2 transcripts, relative to untreated control cells [289]. Laying hens fed on a diet supplemented with 0.5% dry mulberry leaves (ML) for 12 weeks were characterised by significantly increased mRNA levels of antioxidant-regulated genes, including *Nrf2, HO-1* and *GST* in peripheral blood mononuclear cells in comparison to the birds fed on the control diet [290]. Furthermore, the serum MDA concentration was shown to be decreased and the catalase and SOD activities increased in all the ML-supplemented groups in comparison to the control group birds. Niu et al. [291] investigated the effect of dietary supplementation of fermented ginkgo biloba leaves (FGBLs) on nutrient utilization, intestinal digestive function and antioxidant activity of broilers. It was shown that chickens fed with a 3.5 g/kg FGBL diet were characterised by increased body weight gain, feed intake and relative weight of duodenum. At the same time, antioxidant and digestive enzyme activities were increased and MDA concentration decreased in the pancreas and small intestine of the experimental birds. This was accompanied by upregulation of *Nrf2* in the small intestine [291]. Sahin et al. [292] evaluated genistein in hens and showed it prevented the development of spontaneous ovarian cancer and inhibited tumour growth. This was associated with reduction of serum MDA and downregulation of the expression of NF- κB and Bcl-2 and upregulation of the expression of Nrf2, HO-1 and Bax at the protein level in ovarian tissues. There was a significant linear effect of dietary curcumin on the relative abundance of SOD1, GPx1, CAT, HO-1 and Nrf2 transcripts and a quadratic increase in the activities of GPx and T-AOC in jejunal mucosa of growing ducklings [293]. Although phytochemical supplementation of poultry diets is a relatively new area of research, the former work points to the synergetic benefits in the avian antioxidant defence system.

### 7.8. Other Nutrients and Probiotics

Dietary tryptophan (0.08–0.16%) was shown to decrease GSH and the GSH/GSSG ratio in plasma and increase *Nrf2* and *TNF-α* gene expression in the ileal mucosa of Chinese yellow-feathered broiler breeder hens [294]. Dietary methionine (Met) levels (2.00, 2.75, 3.50, 4.25, 5.00 or 5.75 g/kg for 24 weeks) showed a linear and quadratic effect on the gene expression of *GPx1*, *HO-1* and *Nrf2*, and quadratically increased the activity of GPx and total antioxidant capacity (T-AOC) in the liver of duck breeders. Furthermore, maternal dietary Met enhanced the gene expression of *GPx1*, *HO-1* and *Nrf2*, increased activity of GPX and T-AOC and reduced carbonylated protein in the brains of hatchlings [295]. Dietary probiotic *Bacillus subtilis* (Strain fmbj) was shown to increases antioxidant capacity and oxidative stability of chicken breast muscle during storage. This was associated with increased mRNA expression of antioxidant genes (*Nrf2, HO-1, SOD, CAT, GPx*) and decreased the oxidative damage index (MDA, ROS, PC, 8-OhdG) in chicken breast muscle [296] and expression of Nrf2, HO-1, SOD and GPx in chicken liver mitochondria due to the dietary probiotic [297]. Furthermore, there is a range of other nutrients upregulating *Nrf2* expression in different poultry tissues, including carnitine [90], taurine [298] and silymarin [91].

**Figure 4:**
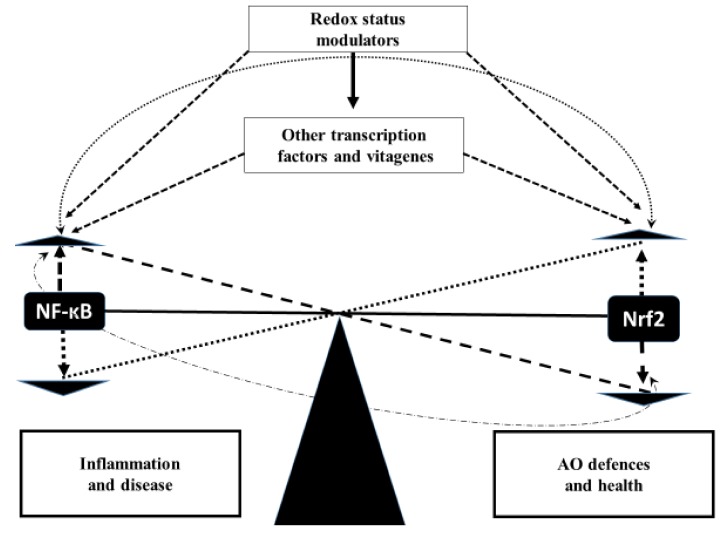
Hypothetical scheme of Nrf2–NF-κB crosstalk.

Therefore, the aforementioned data have shown that Nrf2 activation is an adaptive mechanism to deal with various stressors in poultry. Indeed, by improving ROS scavenging and restoring redox homeostasis in stress conditions, Nrf2 can prevent/decrease stress-related detrimental changes in poultry and play a crucial role in anti-stress strategy development. However, when stress is excessive and Nrf2 expression is decreased, oxidative stress occurs leading to detrimental consequences in terms of the productive and reproductive performance of poultry. There is a range of nutrients, including various phytochemicals, carnitine, taurine and silymarin, that are able to prevent the detrimental effects of stressors on Nrf2 expression. Furthermore, interest in this transcription factor goes beyond stress. For example, when a comparison of a single male modern broiler line compared to a foundational Barred Plymouth Rock chicken line was conducted, it showed that Nrf2 expression in breast muscle was much higher in the modern broiler compared to the foundational BPR line [299], and it was suggested Nrf2-mediated oxidative stress response pathways are involved in breast muscle growth in chickens [300]. In recent years, several compounds, including carnitine, have been shown to have inhibitory activities against multiple components of the NF-κB activation pathway. Transcription factors Nrf2, NF-κB and HSF1 enable the eukaryotic cell to adapt to various forms of oxidative, electrophilic, thermal, inflammatory and other stressors by orchestrating elaborate transcriptional programs termed the Keap1/Nrf2 pathway, NF-κB/IκB pathway and the heat shock response [253]. In general, induction of these programs is associated with upregulation of various protective mechanisms including molecular chaperones, antioxidant and drug-metabolizing enzymes, proteins responsible for the repair and clearance of damaged macromolecules as well as for the maintenance of cell structure, redox and intermediary metabolism [253]. It is clear that Nrf2 and NF-κB have interactive expression and activity to coordinate anti-oxidative and inflammatory responses, but it is not yet known how this interconnection takes place [301]. Thus, stress-associated changes in redox balance and in activities of transcription factors such as Nrf2/Keap1 and NF-κB/ IκB/IKK provide adaptive cell responses to oxidants and a variety of stress stimuli through regulation of gene expression under both physiological and pathological conditions [302]. Despite the accepted concept of physiological ROS/RNS signalling, there is still no complete consensus on molecular mechanisms explaining the beneficial or deleterious effects of ROS on biomolecules and cellular functions [302]. The hypothetical scheme of Nrf2–NF-κB cross-talk is shown in Figure 4.

There is a delicate balance between Nrf2 and NF-κB expression in various tissues and, in physiological conditions, the balance is well maintained. It seems likely that increased NF-κB expression due to the presence of moderate stressors can cause simultaneous compensatory increases in expression of Nrf2 leading to improved antioxidant defences and decreased NF-κB expression as a feedback mechanism. However, when stress is too high, this compensatory adaptive mechanism will not be effective, and an increased NF-κB expression will be associated with a decreased Nrf2 expression. Therefore, once the ability to balance AO defences against ROS production is overwhelmed due to the extremely high stress, redox status will be altered, resulting in an inhibited Nrf2/NF-κB balance which leads to detrimental consequences in terms of health (immunosuppression), productive and reproductive performance in poultry. Importantly, other transcription factors and vitagenes are also involved in regulation of the balance.

## 8. Conclusions

Commercial poultry production is associated with a range of stressors, from hatching (high temperature and humidity) up to slaughter (catching, transportation and holding). In many cases, it is possible to improve the rearing and welfare conditions of broilers during rearing, but the major limitation is the cost of such improvements. A growing body of information clearly indicates that an excess of ROS/RNS production and oxidative stress are major detrimental consequences of most common commercial stressors in poultry production. During evolution, antioxidant defence systems developed in poultry to survive in an oxygenated atmosphere. They include a complex network of internally synthesised (e.g., antioxidant enzymes, GSH, CoQ) and externally supplied (vitamin E, carotenoids, etc.) antioxidants. In fact, all antioxidants in the body are working cooperatively as a team to maintain optimal redox balance in the cell/body. This balance is a key element in providing the necessary conditions for cell signalling, a vital process for regulation of the expression of various genes, stress adaptation and homeostasis maintenance in birds. Since ROS/RNS are considered to be important signalling molecules, their concentration is strictly regulated by the antioxidant defence network in conjunction with various transcription factors and vitagenes. Therefore, activation of such transcription factors as Nrf2 leads to an additional synthesis of an array of protective molecules which can deal with increased ROS/RNS production. However, when stress is too high, leading to a free radical concentration higher than the threshold for cells/tissues, other transcription factors including NF-κB become predominant, and inflammation and apoptosis predispose healthy tissues to damage, leading to the development of various disease states and decreasing the productive and reproductive performances of poultry. The Nrf2 pathway is shown to play a vital role in health resilience and can be made more robust and responsive by certain dietary factors including various phytochemicals and trace minerals [303]. Therefore, it is a challenging task to develop a system of optimal antioxidant supplementation to help growing/productive birds maintain effective antioxidant defences and redox balance in the body. On the one hand, antioxidants, such as vitamin E, or minerals, such as Se (a precursor of GPx and other selenoproteins), Mn, Cu and Zn (important parts of SOD), have become a compulsory part of the commercial premixes for poultry and, in most cases, their levels in premixes are sufficient to meet the physiological requirements in these elements independently on their provision with feed ingredients. On the other hand, regarding the aforementioned commercially relevant stressors, there is a need for additional support for the antioxidant system in poultry. Current research is concentrated on the usage of an optimal dietary Se form [60] or increasing vitamin E supplementation [86]. There are also numerous attempts to use various phytochemicals in poultry diets, but their success is quite variable due to the low rate of the active compounds’ absorption and assimilation [286]. The new direction in improving the antioxidant defences of poultry in stress conditions is related to an opportunity to activate a range of vitagenes (via Nrf2-related mechanisms: SOD, HO-1, GSH, Trx, or other mechanisms: HSP, sirtuins, etc.) to maximize an internal AO protection and redox balance maintenance. Therefore, the development of vitagene-regulating nutritional supplements is on the agenda of many commercial companies worldwide. One successful example could be a complex mixture of nutrients including carnitine, betaine, vitamins, minerals, organic acids, etc., which are commercially available for poultry [22,105,304,305,306]. Indeed, prevention of the detrimental consequences of stressors and improved performance in broilers [22,303], broiler breeders and layers [305,306] using vitagene-activation may, based on references in this review, optimize the antioxidant defence system. It seems likely that the vitagene concept of fighting stress could be used for maintenance/improvement of eggshell health, gut health and liver health of modern poultry in conditions under stress (for a review, see Reference [105]). Furthermore, improved laboratory techniques used in quantifying avian cellular Nrf2, may lead to further elucidation of the mechanisms in oxidative stress biology.

## Figures and Tables

**Table 1:** Main stressors in poultry production.

Stresses	References
**Technological stressors**	
Chick placement	[1,17,18,19,20,21,22]
Increased stocking density	[23,24,25,26,27,28,29,30,31]
Weighing, grading, group formation, catching, transferring to breeder houses	[1,18,19]
Prolonged egg storage, egg transportation, inadequate egg storage conditions, incorrect incubation regimes	[1,18,19]
**Environmental stressors**	
Inadequate temperature	[32,33,34,35,36,37,38,39]
Inadequate ventilation and increased dust	[1,18,19,40,41]
Inadequate lightning	[1,18,19,42,43]
**Nutritional stressors**	
Mycotoxins	[44,45,46,47,48,49,50]
Oxidised fat	[51,52,53,54,55]
Toxic metals (lead, cadmium, mercury, etc.)	[17,56,57,58,59]
Imbalance of minerals (Se, Zn, Mn, Cu, etc.) and other nutrients	[2,15,18,19,60,61,62,63]
Low water quality	[2,18,19,64,65,66,67]
Usage of coccidiostats and other drugs via feed or water	[2,18,19,68,69]
**Internal stressors**	
Vaccinations	[70,71,72]
Microbial or virus challenges	[73,74,75,76,77,78]
Gut dis-bacteriosis	[79,80,81,82,83]
Pipping and hatching	[5,84,85]

**Table 2:** Major components of the vitagene network (adapted from References [106,108,111,112]).

**Molecular level**	**Cellular level**
AO defence systems	Cell proliferation
DNA-repair systems	Cell differentiation
Genetic information transfer	Cell membrane integrity
Synthesis of stress proteins	Stability of intracellular milieu
Proteasomal function/regulation	Macromolecular turnover regulation
**Tissue and organ level**	**Physiological and redox control level**
Neutralization and removing toxic chemicals	Stress response
Tissue regeneration and wound healing	Hormonal response
Tumour suppression	Immune response
Cell death and cell replacement	Thermoregulation
	Neuronal response

**Table 3:** Principal functions of enzymes encoded by Nrf2 target genes and involved in the antioxidant defence [186,197,202,203,204].

Gene Name	Abbreviation	Enzyme Principal Functions
Superoxide dismutase	*SOD*	Dismutation of superoxide radicals to molecular oxygen and hydrogen peroxide
Glutathione peroxidase	*GPx*	Detoxification of hydrogen peroxide, organic hydroperoxides and lipid peroxides
Glutamate cysteine ligase	*GCL*	Synthesis of GSH (rate-limiting step)
Glutathione reductase	*GR*	Conversion of glutathione disulphide into the reduced glutathione
Glutathione S-transferase	*GST*	Detoxification of xenobiotics and electrophiles by conjugation with GSH
Sulfiredoxin	*SRXN1*	Reduction of cysteine sulfinic acid formed in peroxiredoxins
Catalase	*CAT*	Transformation of H2O2 into water and oxygen
Thioredoxin 1	*Trx*	Reduction of other proteins by cysteine thiol–disulphide exchange
Thioredoxin reductase	*TrxR*	AO defence and maintaining redox balance
Thioredoxin peroxidase (peroxiredoxins)	*PRDX1*	Reduction of hydrogen peroxide and alkyl hydroperoxides
Heme oxygenase 1	*HO-1*	Heme degradation to carbon monoxide
Glucose 6-phosphate dehydrogenase; 6-phosphogluconate dehydrogenase;	*G6PD* *6PGDH*	Generation of NADPH, the critical cofactor fuelling antioxidant reaction
Malic enzyme 1; Isocitrate dehydrogenase 1	*ME1* *IDH1*	
NAD(P)H quinone oxidoreductase-1	*NQO1*	Reduction of quinones to hydroquinones

## Data Availability

Data sharing is not applicable to this article as no datasets were generated or analysed during the current study.

## Abbreviations

AFB1	aflatoxin B1
AO	antioxidant
AP1	transcription factor
AREs	antioxidant-response elements
CoQ	Coenzyme Q
FCR	feed conversion ratio
HSF1	heat shock factor
PGC-1α	peroxisome proliferator-activated receptor-γ coactivator
p53	tumour protein p53
PPAR	peroxisome proliferator-activated receptor
PUFAs	polyunsaturated fatty acids
ROS	reactive oxygen species
RNS	reactive nitrogen species
FoxO	transcription factors
GR	glutathione reductase
Grx	glutaredoxin
GSH	reduced glutathione
GPx	glutathione peroxidase
GST	glutathione S-transferase
HO-1	heme oxygenase 1
HS	heat stress
HSP	heat shock protein
Keap1	Kelch-like erythroid cell-derived protein with CNC homology (ECH)-associated protein 1
LPS	lipopolysaccharide
MAPK	mitogen-activated protein kinase
MDA	malondialdehyde
Msr	methionine sulfoxide reductase
NF-κB	nuclear factor-kB
NQO1	NAD(P)H:quinone dehydrogenase 1
Nrf2	nuclear factor-erythroid-2- (NF-E2-) and related factor 2
SOD	superoxide dismutase
TN	thermoneutral
TNF-α	tumour necrosis factor
Trx	thioredoxin
TrxR	thioredoxin reductase

## References

[1] Surai P.F., Fisinin V.I. (2016). Vitagenes in poultry production. Part 1. Technological and environmental stresses. Worlds Poult. Sci. J..

[2] Surai P.F., Fisinin V.I. (2016). Vitagenes in poultry production. Part 2. Nutritional and internal stresses. Worlds Poult. Sci. J..

[3] Chen X., Li S., Liu L. (2014). Engineering redox balance through cofactor systems. Trends Biotechnol..

[4] Corsello T., Komaravelli N., Casola A. (2018). Role of Hydrogen Sulfide in NRF2- and Sirtuin-Dependent Maintenance of Cellular Redox Balance. Antioxidants.

[5] Surai P.F. (1999). Vitamin E in avian reproduction. Poult. Avian Biol. Rev..

[6] Surai P.F. (2002). Natural Antioxidants in Avian Nutrition and Reproduction.

[7] Surai P.F. (2006). Selenium in Nutrition and Health.

[8] Santoro M.M. (2018). Fashioning blood vessels by ROS signalling and metabolism. Semin. Cell Dev. Biol..

[9] Moloney J.N., Cotter T.G. (2018). ROS signalling in the biology of cancer. Semin. Cell Dev. Biol..

[10] Francois M., Donovan P., Fontaine F. (2018). Modulating transcription factor activity: Interfering with protein-protein interaction networks. Semin. Cell Dev. Biol..

[11] Cuadrado A., Manda G., Hassan A., Alcaraz M.J., Barbas C., Daiber A., Ghezzi P., León R., López M.G., Oliva B. (2018). Transcription Factor NRF2 as a Therapeutic Target for Chronic Diseases: A Systems Medicine Approach. Pharm. Rev..

[12] Calabrese V., Giordano J., Crupi R., Di Paola R., Ruggieri M., Bianchini R., Ontario M.L., Cuzzocrea S., Calabrese E.J. (2017). Hormesis, cellular stress response and neuroinflammation in schizophrenia: Early onset versus late onset state. J. Neurosci. Res..

[13] Surai P.F., Kochish I.I., Fisinin V.I. (2017). Antioxidant systems in poultry biology: Nutritional modulation of vitagenes. Eur. J. Poult. Sci..

[14] Bureau C., Hennequet-Antier C., Couty M., Guémené D. (2009). Gene array analysis of adrenal glands in broiler chickens following ACTH treatment. Bmc Genom..

[15] Surai P.F. (2018). Selenium in Poultry Nutrition and Health.

[16] Soleimani A.F., Zulkifli I., Omar A.R., Raha A.R. (2011). Physiological responses of 3 chicken breeds to acute heat stress. Poult. Sci..

[17] Surai P.F., Fisinin V.I. (2015). Antioxidant-Prooxidant Balance in the Intestine: Applications in Chick Placement and Pig Weaning. J. Vet. Sci. Med..

[18] Fisinin V.I., Surai P.F. (2012). First days of chicken life: From a protection against stresses to an effective adaptation. Russian Poult. Sci. (Ptitsevodstvo Russia).

[19] Fisinin V.I., Surai P.F. (2012). Early chicken nutrition and muscle tissue development. Russian Poult. Sci. (Ptitsevodstvo Russia).

[20] Geyra A., Uni Z., Sklan D. (2001). The effect of fasting at different ages on growth and tissue dynamics in the small intestine of the young chick. Br. J. Nutr..

[21] Karadas F., Surai P.F., Sparks N.H. (2011). Changes in broiler chick tissue concentrations of lipid-soluble antioxidants immediately post-hatch. Comp. Biochem. Physiol. A Mol. Integr. Physiol..

[22] Grigorieva M.A., Velichko O.A., Shabaldin S.V., Fisinin V.I., Surai P.F. (2017). Vitagene regulation as a new strategy to fight stresses in poultry production. Agric. Biol. (Sel’skokhozyaistvennaya Biologiya).

[23] Puron D., Santamaria R., Segura J.C., Alamilla J.L. (1995). Broiler performance at different stocking densities. J. Appl. Poult. Res..

[24] Tsiouris V., Georgopoulou I., Batzios C., Pappaioannou N., Ducatelle R., Fortomaris P. (2015). High stocking density as a predisposing factor for necrotic enteritis in broiler chicks. Avian Pathol..

[25] Simitzis P.E., Kalogeraki E., Goliomytis M., Charismiadou M.A., Triantaphyllopoulos K., Ayoutanti A., Niforou K., Hager-Theodorides A.L., Deligeorgis S.G. (2012). Impact of stocking density on broiler growth performance, meat characteristics, behavioural components and indicators of physiological and oxidative stress. Brit. Poult. Sci..

[26] Sørensen P., Su G., Kestin S.C. (2000). Effects of age and stocking density on leg weakness in broiler chickens. Poult. Sci..

[27] Buijs S., Van Poucke E., Van Dongen S., Lens L., Baert J., Tuyttens F.A. (2012). The influence of stocking density on broiler chicken bone quality and fluctuating asymmetry. Poult. Sci..

[28] Cengiz Ö., Köksal B.H., Tatlı O., Sevim Ö., Ahsan U., Üner A.G., Ulutaş P.A., Beyaz D., Büyükyörük S., Yakan A. (2015). Effect of dietary probiotic and high stocking density on the performance, carcass yield, gut microflora, and stress indicators of broilers. Poult. Sci..

[29] Tong H.B., Lu J., Zou J.M., Wang Q., Shi S.R. (2012). Effects of stocking density on growth performance, carcass yield, and immune status of a local chicken breed. Poult. Sci..

[30] Mirfendereski E., Jahanian R. (2015). Effects of dietary organic chromium and vitamin C supplementation on performance, immune responses, blood metabolites, and stress status of laying hens subjected to high stocking density. Poult. Sci..

[31] Thiamhirunsopit K., Phisalaphong C., Boonkird S., Kijparkorn S. (2014). Effect of chili meal (Capsicum frutescens LINN.) on growth performance, stress index, lipid peroxidation and ileal nutrient digestibility in broilers reared under high stocking density condition. Anim. Feed Sci. Technol..

[32] Lara L., Rostagno M. (2013). Impact of heat stress on poultry production. Animals.

[33] Quinteiro-Filho W.M., Ribeiro A., Ferraz-de-Paula V., Pinheiro M.L., Sakai M., Sá L.R.M., Ferreira A.J., Palermo-Neto J. (2010). Heat stress impairs performance parameters, induces intestinal injury, and decreases macrophage activity in broiler chickens. Poult. Sci..

[34] Song J., Xiao K., Ke Y.L., Jiao L.F., Hu C.H., Diao Q.Y., Shi B., Zou X.T. (2014). Effect of a probiotic mixture on intestinal microflora, morphology, and barrier integrity of broilers subjected to heat stress. Poult. Sci..

[35] Chen X.Y., Li R., Geng Z.Y. (2015). Cold stress initiates the Nrf2/UGT1A1/L-FABP signaling pathway in chickens. Poult. Sci..

[36] Hu R., He Y., Arowolo M.A., Wu S., He J. (2019). Polyphenols as Potential Attenuators of Heat Stress in Poultry Production. Antioxidants.

[37] Nawab A., Ibtisham F., Li G., Kieser B., Wu J., Liu W., Zhao Y., Nawab Y., Li K., Xiao M. (2018). Heat stress in poultry production: Mitigation strategies to overcome the future challenges facing the global poultry industry. J. Therm. Biol..

[38] Farag M.R., Alagawany M. (2018). Physiological alterations of poultry to the high environmental temperature. J. Biol..

[39] Habibian M., Sadeghi G., Ghazi S., Moeini M.M. (2015). Selenium as a feed supplement for heat-stressed poultry: A review. Biol. Trace Elem. Res..

[40] Bottje W.G., Wideman R.F. (1995). Potential role of free radicals in the pathogenesis of pulmonary hypertension syndrome. Poult. Avian Biol. Rev..

[41] Bottje W.G., Wang S., Kelly F.J., Dunster C., Williams A., Mudway I. (1998). Antioxidant defenses in lung lining fluid of broilers: Impact of poor ventilation conditions. Poult. Sci..

[42] Huth J.C., Archer G.S. (2015). Comparison of Two LED Light Bulbs to a Dimmable CFL and their Effects on Broiler Chicken Growth, Stress, and Fear. Poult. Sci..

[43] Van der Pol C.W., Molenaar R., Buitink C.J., Van Roovert-Reijrink I.A., Maatjens C.M., Van den Brand H., Kemp B. (2015). Lighting schedule and dimming period in early life: Consequences for broiler chicken leg bone development. Poult. Sci..

[44] Surai P.F., Dvorska J.E., Diaz D.E. (2005). Effects of Mycotoxins on Antioxidant Status and Immunity. The Mycotoxin Blue Book.

[45] Surai P.F., Mezes M., Melnichuk S.D., Fotina T.I. (2008). Mycotoxins and animal health: From oxidative stress to gene expression. Krmiva.

[46] Tao Y., Xie S., Xu F., Liu A., Wang Y., Chen D., Pan Y., Huang L., Peng D., Wang X. (2018). Ochratoxin A: Toxicity, oxidative stress and metabolism. Food Chem. Toxicol..

[47] Wang X., Wu Q., Wan D., Liu Q., Chen D., Liu Z., Martínez-Larrañaga M.R., Martínez M.A., Anadón A., Yuan Z. (2016). Fumonisins: Oxidative stress-mediated toxicity and metabolism in vivo and in vitro. Arch. Toxicol..

[48] Wu Q.H., Wang X., Yang W., Nüssler A.K., Xiong L.Y., Kuča K., Dohnal V., Zhang X.J., Yuan Z.H. (2014). Oxidative stress-mediated cytotoxicity and metabolism of T-2 toxin and deoxynivalenol in animals and humans: An update. Arch. Toxicol.

[49] Murugesan G.R., Ledoux D.R., Naehrer K., Berthiller F., Applegate T.J., Grenier B., Phillips T.D., Schatzmayr G. (2015). Prevalence and effects of mycotoxins on poultry health and performance, and recent development in mycotoxin counteracting strategies. Poult. Sci..

[50] Kövesi B., Cserháti M., Erdélyi M., Zándoki E., Mézes M., Balogh K. (2019). Long-Term Effects of Ochratoxin A on the Glutathione Redox System and Its Regulation in Chicken. Antioxidants.

[51] Tavárez M.A., Boler D.D., Bess K.N., Zhao J., Yan F., Dilger A.C., McKeith F.K., Killefer J. (2011). Effect of antioxidant inclusion and oil quality on broiler performance, meat quality, and lipid oxidation. Poult. Sci..

[52] Yue H.Y., Wang J., Qi X.L., Ji F., Liu M.F., Wu S.G., Zhang H.J., Qi G.H. (2011). Effects of dietary oxidized oil on laying performance, lipid metabolism, and apolipoprotein gene expression in laying hens. Poult. Sci.

[53] Zhang W., Xiao S., Lee E.J., Ahn D.U. (2011). Consumption of oxidized oil increases oxidative stress in broilers and affects the quality of breast meat. J. Agric. Food Chem..

[54] Delles R.M., Xiong Y.L., True A.D., Ao T., Dawson K.A. (2014). Dietary antioxidant supplementation enhances lipid and protein oxidative stability of chicken broiler meat through promotion of antioxidant enzyme activity. Poult. Sci..

[55] Delles R.M., Xiong Y.L., True A.D., Ao T., Dawson K.A. (2015). Augmentation of water-holding and textural properties of breast meat from oxidatively stressed broilers by dietary antioxidant regimens. Brit. Poult. Sci..

[56] Pappas A.C., Zoidis E., Georgiou C.A., Demiris N., Surai P.F., Fegeros K. (2011). Influence of organic selenium supplementation on the accumulation of toxic and essential trace elements involved in the antioxidant system of chicken. Food Addit. Contam. Part A Chem. Anal. Control Expo. Risk Assess..

[57] Guo Q., Majeed S., Xu R., Zhang K., Kakade A., Khan A., Hafeez F.Y., Mao C., Liu P., Li X. (2019). Heavy metals interact with the microbial community and affect biogas production in anaerobic digestion: A review. J. Environ. Manag.

[58] Kar I., Mukhopadhayay S.K., Patra A.K., Pradhan S. (2018). Bioaccumulation of selected heavy metals and histopathological and hematobiochemical alterations in backyard chickens reared in an industrial area, India. Environ. Sci. Pollut. Res. Int..

[59] Bao R.K., Zheng S.F., Wang X.Y. (2017). Selenium protects against cadmium-induced kidney apoptosis in chickens by activating the PI3K/AKT/Bcl-2 signaling pathway. Environ. Sci. Pollut. Res. Int..

[60] Surai P.F., Kochish I.I. (2018). Nutritional modulation of the antioxidant capacities in poultry: The case of selenium. Poult. Sci..

[61] Yao L., Du Q., Yao H., Chen X., Zhang Z., Xu S. (2015). Roles of oxidative stress and endoplasmic reticulum stress in selenium deficiency-induced apoptosis in chicken liver. Biometals.

[62] Cinar M., Yildirim E., Yigit A.A., Yalcinkaya I., Duru O., Kisa U., Atmaca N. (2014). Effects of dietary supplementation with vitamin C and vitamin E and their combination on growth performance, some biochemical parameters, and oxidative stress induced by copper toxicity in broilers. Biol. Trace Elem. Res..

[63] Berzina N., Markovs J., Dizhbite T., Apsite M., Vasilyeva S., Basova N., Smirnova G., Isajevs S. (2013). Oxidative stress and innate immunity status in chickens exposed to high dose of ascorbic acid. Cell Biochem. Funct..

[64] Maes S., Vackier T., Huu S.N., Heyndrickx M., Steenackers H., Sampers I., Raes K., Verplaetse A., De Reu K. (2019). Occurrence and characterisation of biofilms in drinking water systems of broiler houses. BMC Microbiol..

[65] Rauch E., Hirsch N., Firnkäs N., Erhard M.H., Bergmann S. (2016). Animal hygiene, water quality and animal health using round drinkers as an animal-friendly water supply for Pekin ducks under practical conditions. Berl Munch Tierarztl Wochenschr..

[66] Giammarino M., Quatto P. (2015). Nitrates in drinking water: Relation with intensive livestock production. J. Prev. Med. Hyg..

[67] King A.J. (1996). Water quality and poultry production. Poult Sci..

[68] Charvat R.A., Arrizabalaga G. (2016). Oxidative stress generated during monensin treatment contributes to altered Toxoplasma gondii mitochondrial function. Sci. Rep..

[69] Yu S.N., Kim S.H., Kim K.Y., Ji J.H., Seo Y.K., Yu H.S., Ahn S.C. (2017). Salinomycin induces endoplasmic reticulum stress-mediated autophagy and apoptosis through generation of reactive oxygen species in human glioma U87MG cells. Oncol. Rep..

[70] Yang X.J., Li W.L., Feng Y., Yao J.H. (2011). Effects of immune stress on growth performance, immunity, and cecal microflora in chickens. Poult. Sci..

[71] Nelson J.R., McIntyre D.R., Pavlidis H.O., Archer G.S. (2018). Reducing Stress Susceptibility of Broiler Chickens by Supplementing a Yeast Fermentation Product in the Feed or Drinking Water. Animals.

[72] Kaab H., Bain M.M., Eckersall P.D. (2018). Acute phase proteins and stress markers in the immediate response to a combined vaccination against Newcastle disease and infectious bronchitis viruses in specific pathogen free (SPF) layer chicks. Poult. Sci..

[73] Rehman Z.U., Meng C., Sun Y., Safdar A., Pasha R.H., Munir M., Ding C. (2018). Oxidative Stress in Poultry: Lessons from the Viral Infections. Oxid. Med. Cell. Longev..

[74] Rehman Z.U., Che L., Ren S., Liao Y., Qiu X., Yu S., Sun Y., Tan L., Song C., Liu W. (2018). Supplementation of Vitamin E Protects Chickens from Newcastle Disease Virus-Mediated Exacerbation of Intestinal Oxidative Stress and Tissue Damage. Cell Physiol. Biochem..

[75] Rehman Z.U., Qiu X., Sun Y., Liao Y., Tan L., Song C., Yu S., Ding Z., Munir M., Nair V. (2018). Vitamin E Supplementation Ameliorates Newcastle Disease Virus-iduced Oxidative Stress and Alleviates Tissue Damage in the Brains of Chickens. Viruses.

[76] Zhao D., Yang J., Han K., Liu Q., Wang H., Liu Y., Huang X., Zhang L., Li Y. (2019). The unfolded protein response induced by Tembusu virus infection. BMC Vet. Res..

[77] Neerukonda S.N., Katneni U.K., Bott M., Golovan S.P., Parcells M.S. (2018). Induction of the unfolded protein response (UPR) during Marek’s disease virus (MDV) infection. Virology.

[78] Da Rosa G., Da Silva A.S., Souza C.F., Baldissera M.D., Mendes R.E., Araujo D.N., Alba D.F., Boiago M.M., Stefani L.M. (2019). Impact of colibacillosis on production in laying hens associated with interference of the phosphotransfer network and oxidative stress. Microb. Pathog..

[79] He J., He Y., Pan D., Cao J., Sun Y., Zeng X. (2019). Associations of Gut Microbiota with Heat Stress-Induced Changes of Growth, Fat Deposition, Intestinal Morphology, and Antioxidant Capacity in Ducks. Front. Microbiol..

[80] Le Roy C.I., Woodward M.J., Ellis R.J., La Ragione R.M., Claus S.P. (2019). Antibiotic treatment triggers gut dysbiosis and modulates metabolism in a chicken model of gastro-intestinal infection. BMC Vet. Res..

[81] Pereira R., Bortoluzzi C., Durrer A., Fagundes N.S., Pedroso A.A., Rafael J.M., Perim J.E.L., Zavarize K.C., Napty G.S., Andreote F.D. (2019). Performance and intestinal microbiota of chickens receiving probiotic in the feed and submitted to antibiotic therapy. J. Anim. Physiol. Anim. Nutr..

[82] Ducatelle R., Goossens E., De Meyer F., Eeckhaut V., Antonissen G., Haesebrouck F., Van Immerseel F. (2018). Biomarkers for monitoring intestinal health in poultry: Present status and future perspectives. Vet. Res..

[83] Janssens Y., Nielandt J., Bronselaer A., Debunne N., Verbeke F., Wynendaele E., Van Immerseel F., Vandewynckel Y.P., De Tré G., De Spiegeleer B. (2018). Disbiome database: Linking the microbiome to disease. BMC Microbiol..

[84] Surai P.F., Fisinin V.I. (2013). Natural antioxidants in chicken embryogenesis and protection against stresses in postnatal development. Agric. Biol..

[85] Surai P.F., Fisinin V.I., Karadas F. (2016). Antioxidant Systems in Chick Embryo Development. Part 1. Vitamin E, Carotenoids and Selenium. Anim. Nutr..

[86] Surai P.F., Kochish I.I., Romanov M.N., Griffin D.K. (2019). Nutritional modulation of the antioxidant capacities in poultry: The case of vitamin E. Poult Sci..

[87] Skulachev V.P. (1998). Biochemical mechanisms of evolution and the role of oxygen. Biochemistry.

[88] Surai P.F. (2015). Antioxidant Action of Carnitine: Molecular Mechanisms and Practical Applications. EC Vet. Sci..

[89] Surai P.F. (2015). Carnitine Enigma: From Antioxidant Action to Vitagene Regulation. Part 1. Absorption, Metabolism and Antioxidant Activities. J. Veter. Sci. Med..

[90] Surai P.F. (2015). Carnitine Enigma: From Antioxidant Action to Vitagene Regulation Part 2. Transcription Factors and Practical Applications. J. Veter. Sci. Med..

[91] Surai P.F. (2015). Silymarin as a Natural Antioxidant: An Overview of the Current Evidence and Perspectives. Antioxidants.

[92] Surai P.F. (2015). Antioxidant systems in Poultry Biology: Heat shock proteins. J. Sci..

[93] Surai P.F. (2016). Antioxidant systems in Poultry Biology: Superoxide dismutase. Anim. Nutr..

[94] Surai P.F. (2017). Antioxidant defences: Food for thoughts. EC Nutr..

[95] Sies H., Sies H. (1985). Oxidative stress: Introductory remarks. Oxidative Stress.

[96] Sies H. (2015). Oxidative stress: A concept in redox biology and medicine. Redox. Biol..

[97] Sies H., Berndt C., Jones D.P. (2017). Oxidative stress. Annu. Rev. Biochem..

[98] Sies H. (2018). On the history of oxidative stress: Concept and some aspects of current development. Curr. Opin. Toxicol..

[99] Sies H., Fink G. (2019). Oxidative Stress: Eustress and Distress in Redox Homeostasis. Stress: Physiology, Biochemistry, and Pathology.

[100] Reczek C.R., Chandel N.S. (2015). ROS-dependent signal transduction. Curr. Opin. Cell Biol..

[101] Niki E. (2014). Antioxidants: Basic principles, emerging concepts, and problems. Biomed J..

[102] Pomatto L.C.D., Davies K.J.A. (2018). Adaptive homeostasis and the free radical theory of ageing. Free Radic. Biol. Med..

[103] Forman H.J. (2016). Redox signaling: An evolution from free radicals to aging. Free Radic. Biol. Med..

[104] Yan L.J. (2014). Positive oxidative stress in aging and aging-related disease tolerance. Redox Biol..

[105] Surai P.F., Kochish I.I., Fisinin V.I., Grozina A.A., Shatskikh E.V. (2018). Molecular Mechanisms of Gut Health Support in Poultry: Role of Microbiota.

[106] Rattan S.I. (1998). The nature of gerontogenes and vitagenes. Antiaging effects of repeated heat shock on human fibroblasts. Ann. N. Y. Acad. Sci..

[107] Calabrese V., Boyd-Kimball D., Scapagnini G., Butterfield D.A. (2004). Nitric oxide and cellular stress response in brain aging and neurodegenerative disorders: The role of vitagenes. In Vivo.

[108] Calabrese V., Guagliano E., Sapienza M., Panebianco M., Calafato S., Puleo E., Pennisi G., Mancuso C., Butterfield D.A., Stella A.G. (2007). Redox regulation of cellular stress response in aging and neurodegenerative disorders: Role of vitagenes. Neurochem. Res..

[109] Calabrese V., Cornelius C., Mancuso C., Barone E., Calafato S., Bates T., Rizzarelli E., Kostova A.T. (2009). Vitagenes, dietary antioxidants and neuroprotection in neurodegenerative diseases. Front. Biosci..

[110] Calabrese V., Scapagnini G., Davinelli S., Koverech G., Koverech A., De Pasquale C., Salinaro A.T., Scuto M., Calabrese E.J., Genazzani A.R. (2014). Sex hormonal regulation and hormesis in aging and longevity: Role of vitagenes. J. Cell Commun. Signal..

[111] Surai P.F., Fisinin V.I. (2016). Vitagenes in poultry production. Part 3. Vitagene concept development. Worlds Poult. Sci. J..

[112] Surai P.F., Fisinin V.I., Watson R.R., De Meester F. (2016). Antioxidant system regulation: From vitamins to vitagenes. Handbook of Cholesterol.

[113] Surai P.F., Kochish I.I., Asea Alexzander A.A., Punit K. (2017). Antioxidant systems and vitagenes in poultry biology: Heat Shock Proteins. Heat Shock Proteins in Veterinary.

[114] Pockley A.G., Multhoff G. (2008). Cell stress proteins in extracellular fluids: Friend or foe?. Novartis Found. Symp..

[115] Velichko A.K., Markova E.N., Petrova N.V., Razin S.V., Kantidze O.L. (2013). Mechanisms of heat shock response in mammals. Cell. Mol. Life Sci..

[116] Meijering R.A., Henning R.H., Brundel B.J. (2015). Reviving the protein quality control system: Therapeutic target for cardiac disease in the elderly. Trends Cardiovasc. Med..

[117] Fujimoto M., Nakai A. (2010). The heat shock factor family and adaptation to proteotoxic stress. FEBS J..

[118] Sakurai H., Enoki Y. (2010). Novel aspects of heat shock factors: DNA recognition, chromatin modulation and gene expression. FEBS J..

[119] Takii R., Fujimoto M., Tan K., Takaki E., Hayashida N., Nakato R., Shirahige K., Nakai A. (2015). ATF1 modulates the heat shock response by regulating the stress-inducible heat shock factor 1 transcription complex. Mol. Cell. Biol..

[120] Nakai A., Morimoto R.I. (1993). Characterization of a novel chicken heat shock transcription factor, heat shock factor 3, suggests a new regulatory pathway. Mol. Cell. Biol..

[121] Tanabe M., Nakai A., Kawazoe Y., Nagata K. (1997). Different thresholds in the responses of two heat shock transcription factors, HSF1 and HSF3. J. Biol. Chem..

[122] Inouye S., Katsuki K., Izu H., Fujimoto M., Sugahara K., Yamada S., Shinkai Y., Oka Y., Katoh Y., Nakai A. (2003). Activation of heat shock genes is not necessary for protection by heat shock transcription factor 1 against cell death due to a single exposure to high temperatures. Mol. Cell Biol..

[123] Nakai A., Ishikawa T. (2000). A nuclear localization signal is essential for stress-induced dimer-to-trimer transition of heat shock transcription factor 3. J. Biol. Chem..

[124] Nakai A., Ishikawa T. (2001). Cell cycle transition under stress conditions controlled by vertebrate heat shock factors. Embo J..

[125] Shabtay A., Arad Z. (2006). Reciprocal activation of HSF1 and HSF3 in brain and blood tissues: Is redundancy developmentally related? Am. J. Physiol. Regul. Integr. Comp. Physiol..

[126] Shinkawa T., Tan K., Fujimoto M., Hayashida N., Yamamoto K., Takaki E., Takii R., Prakasam R., Inouye S., Mezger V. (2011). Heat shock factor 2 is required for maintaining proteostasis against febrile-range thermal stress and polyglutamine aggregation. Mol. Biol. Cell.

[127] Vihervaara A., Sistonen L. (2014). HSF1 at a glance. J. Cell. Sci..

[128] Rosenzweig R., Nillegoda N.B., Mayer M.P., Bukau B. (2019). The Hsp70 chaperone network. Nat. Rev. Mol. Cell Biol..

[129] Fernández-Fernández M.R., Valpuesta J.M. (2018). Hsp70 chaperone: A master player in protein homeostasis. F1000Research.

[130] Mayer M.P., Gierasch L.M. (2019). Recent advances in the structural and mechanistic aspects of Hsp70 molecular chaperones. J. Biol. Chem..

[131] Balogi Z., Multhoff G., Jensen T.K., Lloyd-Evans E., Yamashima T., Jäättelä M., Harwood J.L., Vígh L. (2019). Hsp70 interactions with membrane lipids regulate cellular functions in health and disease. Prog. Lipid Res..

[132] Clerico E.M., Meng W., Pozhidaeva A., Bhasne K., Petridis C., Gierasch L.M. (2019). Hsp70 molecular chaperones: Multifunctional allosteric holding and unfolding machines. Biochem. J..

[133] Morimoto R.I., Hunt C., Huang S.Y., Berg K.L., Banerji S.S. (1986). Organization, nucleotide sequence, and transcription of the chicken HSP70 gene. J. Biol. Chem..

[134] Gabriel J.E., Ferro J.A., Stefani R.M., Ferro M.I., Gomes S.L., Macari M. (1996). Effect of acute heat stress on heat shock protein 70 messenger RNA and on heat shock protein expression in the liver of broilers. Br. Poult. Sci..

[135] Leandro N.S., Gonzales E., Ferro J.A., Ferro M.I., Givisiez P.E., Macari M. (2004). Expression of heat shock protein in broiler embryo tissues after acute cold or heat stress. Mol. Reprod. Dev..

[136] Maamoun H., Benameur T., Pintus G., Munusamy S., Agouni A. (2019). Crosstalk Between Oxidative Stress and Endoplasmic Reticulum (ER) Stress in Endothelial Dysfunction and Aberrant Angiogenesis Associated with Diabetes: A Focus on the Protective Roles of Heme Oxygenase (HO)-1. Front. Physiol..

[137] Lee H., Choi Y.K. (2018). Regenerative Effects of Heme Oxygenase Metabolites on Neuroinflammatory Diseases. Int. J. Mol. Sci..

[138] Sebastián V.P., Salazar G.A., Coronado-Arrázola I., Schultz B.M., Vallejos O.P., Berkowitz L., Álvarez-Lobos M.M., Riedel C.A., Kalergis A.M., Bueno S.M. (2018). Heme Oxygenase-1 as a Modulator of Intestinal Inflammation Development and Progression. Front. Immunol..

[139] Waza A.A., Hamid Z., Ali S., Bhat S.A., Bhat M.A. (2018). A review on heme oxygenase-1 induction: Is it a necessary evil. Inflamm. Res..

[140] Bonkovsky H.L., Healey J.F., Pohl J. (1990). Purification and characterization of heme oxygenase from chick liver. Comparison of the avian and mammalian enzymes. Eur. J. Biochem..

[141] Druyan S., Cahaner A., Ashwell C.M. (2007). The expression patterns of hypoxia-inducing factor subunit alpha-1, heme oxygenase, hypoxia upregulated protein 1, and cardiac troponin T during development of the chicken heart. Poult. Sci..

[142] Surai P.F., Noble R.C., Speake B.K. (1996). Tissue-specific differences in antioxidant distribution and susceptibility to lipid peroxidation during development of the chick embryo. Biochim. Biophys. Acta..

[143] Halliwell B. (1994). Free radicals and antioxidants: A personal view. Nutr. Rev..

[144] McCord J.M., Fridovich I. (1969). Superoxide dismutase: An enzymatic function for erythrocuprein (hemocuprein). J. Biol. Chem..

[145] Azadmanesh J., Borgstahl G.E.O. (2018). A Review of the Catalytic Mechanism of Human Manganese Superoxide Dismutase. Antioxidants.

[146] Weisiger R.A., Fridovich I. (1973). Superoxide dismutase. Organelle specificity. J. Biol. Chem..

[147] Surai P.F. (1999). Tissue-specific changes in the activities of antioxidant enzymes during the development of the chicken embryo. Brit. Poult. Sci..

[148] Surai P.F., Blesbois E., Grasseau I., Ghalah T., Brillard J.-P., Wishart G.J., Cerolini S., Sparks N.H. (1998). Fatty acid composition, glutathione peroxidase and superoxide dismutase activity and total antioxidant activity of avian semen. Comp. Biochem. Physiol..

[149] Dali-Youcef N., Lagouge M., Froelich S., Koehl C., Schoonjans K., Auwerx J. (2007). Sirtuins: The ‘magnificent seven’, function, metabolism and longevity. Ann. Med..

[150] Lee S.H., Lee J.H., Lee H.Y., Min K.J. (2019). Sirtuin signaling in cellular senescence and aging. BMB Rep..

[151] Lin S., Xing H., Zang T., Ruan X., Wo L., He M. (2018). Sirtuins in mitochondrial stress: Indispensable helpers behind the scenes. Ageing Res. Rev..

[152] Singh C.K., Chhabra G., Ndiaye M.A., Garcia-Peterson L.M., Mack N.J., Ahmad N. (2018). The Role of Sirtuins in Antioxidant and Redox Signaling. Antioxid. Redox Signal..

[153] Radak Z., Koltai E., Taylor A.W., Higuchi M., Kumagai S., Ohno H., Goto S., Boldogh I. (2013). Redox-regulating sirtuins in aging, caloric restriction, and exercise. Free Radic. Biol. Med..

[154] Lagunas-Rangel F.A. (2019). Current role of mammalian sirtuins in DNA repair. DNA Repair.

[155] Morris B.J. (2013). Seven sirtuins for seven deadly diseases of aging. Free Radic. Biol. Med..

[156] Hubbard B.P., Sinclair D.A. (2014). Small molecule SIRT1 activators for the treatment of aging and age-related diseases. Trends Pharm. Sci..

[157] Nogueiras R., Habegger K.M., Chaudhary N., Finan B., Banks A.S., Dietrich M.O., Horvath T.L., Sinclair D.A., Pfluger P.T., Tschöp M.H. (2012). Sirtuin 1 and sirtuin 3: Physiological modulators of metabolism. Physiol. Rev..

[158] Hickey A.J., Jüllig M., Aitken J., Loomes K., Hauber M.E., Phillips A.R. (2012). Birds and longevity: Does flight driven aerobicity provide an oxidative sink?. Ageing Res. Rev..

[159] Han C., Wan H., Ma S., Liu D., He F., Wang J., Pan Z., Liu H., Li L., He H. (2014). Role of mammalian sirtuin 1 (SIRT1) in lipids metabolism and cell proliferation of goose primary hepatocytes. Mol. Cell. Endocrinol..

[160] Fang X.L., Zhu X.T., Chen S.F., Zhang Z.Q., Zeng Q.J., Deng L., Peng J.L., Yu J.J., Wang L.N., Wang S.B. (2014). Differential gene expression pattern in hypothalamus of chickens during fasting-induced metabolic reprogramming: Functions of glucose and lipid metabolism in the feed intake of chickens. Poult. Sci..

[161] Xue B., Song J., Liu L., Luo J., Tian G., Yang Y. (2017). Effect of epigallocatechin gallate on growth performance and antioxidant capacity in heat-stressed broilers. Arch. Anim. Nutr..

[162] Ren J., Xu N., Ma Z., Li Y., Li C., Wang Y., Tian Y., Liu X., Kang X. (2017). Characteristics of expression and regulation of sirtuins in chicken (*Gallus gallus*). Genome.

[163] Cogburn L.A., Trakooljul N., Chen C., Huang H., Wu C.H., Carré W., Wang X., White H.B. (2018). Transcriptional profiling of liver during the critical embryo-to-hatchling transition period in the chicken (*Gallus gallus*). Bmc Genom..

[164] Trovato A., Cornelius C., Koverech G., Koverech A., Scuto M., Lodato F., Fronte V., Muccilli V., Reibaldi M., Longo A. (2014). Cellular stress response, redox status, and vitagenes in glaucoma: A systemic oxidant disorder linked to Alzheimer’s disease. Front. Pharmacol..

[165] Trovato A., Siracusa R., Di Paola R., Scuto M., Ontario M.L., Bua O., Di Mauro P., Toscano M.A., Petralia C.C.T., Maiolino L. (2016). Redox modulation of cellular stress response and lipoxin A4 expression by *Hericium Erinaceus* in rat brain: Relevance to Alzheimer’s disease pathogenesis. Immun. Ageing.

[166] Trovato A., Siracusa R., Di Paola R., Scuto M., Fronte V., Koverech G., Luca M., Serra A., Toscano M.A., Petralia A. (2016). Redox modulation of cellular stress response and lipoxin A4 expression by Coriolus versicolor in rat brain: Relevance to Alzheimer’s disease pathogenesis. Neurotoxicology.

[167] Calabrese V., Giordano J., Signorile A., Laura Ontario M., Castorina S., De Pasquale C., Eckert G., Calabrese E.J. (2016). Major pathogenic mechanisms in vascular dementia: Roles of cellular stress response and hormesis in neuroprotection. J. Neurosci. Res..

[168] Calabrese V., Giordano J., Ruggieri M., Berritta D., Trovato A., Ontario M.L., Bianchini R., Calabrese E.J. (2016). Hormesis, cellular stress response, and redox homeostasis in autism spectrum disorders. J. Neurosci. Res..

[169] Calabrese V., Calafato S., Puleo E., Cornelius C., Sapienza M., Morganti P., Mancuso C. (2008). Redox regulation of cellular stress response by ferulic acid ethyl ester in human dermal fibroblasts: Role of vitagenes. Clin. Dermatol..

[170] Cornelius C., Trovato Salinaro A., Scuto M., Fronte V., Cambria M.T., Pennisi M., Bella R., Milone P., Graziano A., Crupi R. (2013). Cellular stress response, sirtuins and UCP proteins in Alzheimer disease: Role of vitagenes. Immun. Ageing.

[171] Cornelius C., Koverech G., Crupi R., Di Paola R., Koverech A., Lodato F., Scuto M., Salinaro A.T., Cuzzocrea S., Calabrese E.J. (2014). Osteoporosis and Alzheimer pathology: Role of cellular stress response and hormetic redox signaling in aging and bone remodeling. Front. Pharmacol..

[172] Dattilo S., Mancuso C., Koverech G., Di Mauro P., Ontario M.L., Petralia C.C., Petralia A., Maiolino L., Serra A., Calabrese E.J. (2015). Heat shock proteins and hormesis in the diagnosis and treatment of neurodegenerative diseases. Immun. Ageing.

[173] Calabrese V., Cornelius C., Trovato A., Cavallaro M., Mancuso C., Di Rienzo L., Condorelli D., De Lorenzo A., Calabrese E.J. (2010). The hormetic role of dietary antioxidants in free radical-related diseases. Curr. Pharm. Des..

[174] Calabrese V., Dattilo S., Petralia A., Parenti R., Pennisi M., Koverech G., Calabrese V., Graziano A., Monte I., Maiolino L. (2015). Analytical approaches to the diagnosis and treatment of aging and aging-related disease: Redox status and proteomics. Free Radic. Res..

[175] Calabrese V., Cornelius C., Cuzzocrea S., Iavicoli I., Rizzarelli E., Calabrese E.J. (2011). Hormesis, cellular stress response and vitagenes as critical determinants in aging and longevity. Mol. Asp. Med..

[176] Calabrese V., Cornelius C., Dinkova-Kostova A.T., Iavicoli I., Di Paola R., Koverech A., Cuzzocrea S., Rizzarelli E., Calabrese E.J. (2012). Cellular stress responses, hormetic phytochemicals and vitagenes in aging and longevity. Biochim. Biophys. Acta.

[177] Fisinin V.I., Surai P.F. (2011). Effective protection from stresses in poultry production: From vitamins to vitagenes. Part 1. Ptitza I Ptitzeproducti.

[178] Fisinin V.I., Surai P.F. (2011). Effective protection from stresses in poultry production: From vitamins to vitagenes. Part 2. Ptitza I Ptitzeproducti.

[179] Surai P.F., Fisinin V.I. (2012). Modern methods of fighting stresses in poultry production: From antioxidants to vitagenes. Agricult. Biol..

[180] Calabrese V., Cornelius C., Dinkova-Kostova A.T., Calabrese E.J. (2009). Vitagenes, cellular stress response, and acetylcarnitine: Relevance to hormesis. Biofactors.

[181] Surai P.F. (2018). Taurine and carnitine in poultry production: From vitagene activation to chicken health maintenance. Ukr. Poult. Sci..

[182] Ma Q., He X. (2012). Molecular basis of electrophilic and oxidative defense: Promises and perils of Nrf2. Pharmacol. Rev..

[183] Majzunova M., Dovinova I., Barancik M., Chan J.Y. (2013). Redox signaling in pathophysiology of hypertension. J. Biomed. Sci..

[184] Song P., Zou M.H. (2014). Redox regulation of endothelial cell fate. Cell Mol. Life Sci..

[185] Kweider N., Huppertz B., Kadyrov M., Rath W., Pufe T., Wruck C.J. (2014). A possible protective role of Nrf2 in preeclampsia. Ann. Anat..

[186] Tu W., Wang H., Li S., Liu Q., Sha H. (2019). The Anti-Inflammatory and Anti-Oxidant Mechanisms of the Keap1/Nrf2/ARE Signaling Pathway in Chronic Diseases. Aging Dis..

[187] Marinho H.S., Real C., Cyrne L., Soares H., Antunes F. (2014). Hydrogen peroxide sensing, signaling and regulation of transcription factors. Redox Biol..

[188] Wang X., Hai C. (2016). Novel insights into redox system and the mechanism of redox regulation. Mol. Biol. Rep..

[189] Lushchak V.I. (2011). Adaptive response to oxidative stress: Bacteria, fungi, plants and animals. Comp. Biochem. Physiol. C Toxicol. Pharm..

[190] Itoh K., Mimura J., Yamamoto M. (2010). Discovery of the negative regulator of Nrf2, Keap1: A historical overview. Antioxid. Redox Signal..

[191] Tang W., Jiang Y.F., Ponnusamy M., Diallo M. (2014). Role of Nrf2 in chronic liver disease. World J. Gastroenterol..

[192] Howden R. (2013). Nrf2 and cardiovascular defense. Oxid. Med. Cell Longev..

[193] Vriend J., Reiter R.J. (2015). The Keap1-Nrf2-antioxidant response element pathway: A review of its regulation by melatonin and the proteasome. Mol. Cell. Endocrinol..

[194] Keum Y.S., Choi B.Y. (2014). Molecular and chemical regulation of the Keap1-Nrf2 signaling pathway. Molecules.

[195] Bellezza I., Giambanco I., Minelli A., Donato R. (2018). Nrf2-Keap1 signaling in oxidative and reductive stress. Biochim. Biophys. Acta.

[196] Choi B.H., Kang K.S., Kwak M.K. (2014). Effect of redox modulating NRF2 activators on chronic kidney disease. Molecules.

[197] Helou D.G., Martin S.F., Pallardy M., Chollet-Martin S., Kerdine-Römer S. (2019). Nrf2 Involvement in Chemical-Induced Skin Innate Immunity. Front. Immunol..

[198] Panieri E., Saso L. (2019). Potential Applications of NRF2 Inhibitors in Cancer Therapy. Oxid. Med. Cell. Longev..

[199] Bhakkiyalakshmi E., Sireesh D., Rajaguru P., Paulmurugan R., Ramkumar K.M. (2015). The emerging role of redox-sensitive Nrf2-Keap1 pathway in diabetes. Pharmacol. Res..

[200] Zolnourian A., Galea I., Bulters D. (2019). Neuroprotective Role of the Nrf2 Pathway in Subarachnoid Haemorrhage and Its Therapeutic Potential. Oxid. Med. Cell Longev..

[201] Sussan T.E., Biswal S., Ganguly N.K. (2014). Oxidative stress and respiratory diseases: The critical role of Nrf2. Studies on Respiratory Disorders.

[202] Loboda A., Damulewicz M., Pyza E., Jozkowicz A., Dulak J. (2016). Role of Nrf2/HO-1 system in development, oxidative stress response and diseases: An evolutionarily conserved mechanism. Cell Mol. Life Sci..

[203] Gureev A.P., Shaforostova E.A., Popov V.N. (2019). Regulation of Mitochondrial Biogenesis as a Way for Active Longevity: Interaction Between the Nrf2 and PGC-1α Signaling Pathways. Front. Genet..

[204] Cuadrado A., Rojo A.I., Wells G., Hayes J.D., Cousin S.P., Rumsey W.L., Attucks O.C., Franklin S., Levonen A.L., Kensler T.W. (2019). Therapeutic targeting of the NRF2 and Keap1 partnership in chronic diseases. Nat. Rev. Drug Discov..

[205] Surai P.F., Kochish I.I., Fisinin V.I. (2018). Glutathione peroxidases in poultry biology: Part 1. Classification and mechanisms of action. Worlds Poult. Sci. J..

[206] Surai P.F., Kochish I.I., Fisinin V.I. (2018). Glutathione peroxidases in poultry biology: Part 2. Modulation of enzymatic activities. Worlds Poult. Sci. J..

[207] García-Giménez J.L., Romá-Mateo C., Pérez-Machado G., Peiró-Chova L., Pallardó F.V. (2017). Role of glutathione in the regulation of epigenetic mechanisms in disease. Free Radic. Biol. Med..

[208] Couto N., Wood J., Barber J. (2016). The role of glutathione reductase and related enzymes on cellular redox homoeostasis network. Free Radic. Biol. Med..

[209] Ribas V., García-Ruiz C., Fernández-Checa J.C. (2014). Glutathione and mitochondria. Front. Pharm..

[210] Hansen J.M., Harris C. (2015). Glutathione during embryonic development. Biochim. Biophys. Acta.

[211] Aquilano K., Baldelli S., Ciriolo M.R. (2014). Glutathione: New roles in redox signaling for an old antioxidant. Front. Pharm..

[212] Deponte M. (2013). Glutathione catalysis and the reaction mechanisms of glutathione-dependent enzymes. Biochim. Biophys. Acta.

[213] Griffiths H.R., Dias I.H., Willetts R.S., Devitt A. (2014). Redox regulation of protein damage in plasma. Redox Biol..

[214] Farina M., Aschner M. (2019). Glutathione antioxidant system and methylmercury-induced neurotoxicity: An intriguing interplay. Biochim. Biophys. Acta.

[215] Ren X., Zou L., Zhang X., Branco V., Wang J., Carvalho C., Holmgren A., Lu J. (2017). Redox Signaling Mediated by Thioredoxin and Glutathione Systems in the Central Nervous System. Antioxid. Redox Signal..

[216] Zhang M., An C., Gao Y., Leak R.K., Chen J., Zhang F. (2013). Emerging roles of Nrf2 and phase II antioxidant enzymes in neuroprotection. Prog. Neurobiol..

[217] Hunyadi A. (2019). The mechanism(s) of action of antioxidants: From scavenging reactive oxygen/nitrogen species to redox signaling and the generation of bioactive secondary metabolites. Med. Res. Rev..

[218] Miyazawa T., Burdeos G.C., Itaya M., Nakagawa K., Miyazawa T. (2019). Vitamin E: Regulatory Redox Interactions. Iubmb Life.

[219] Schmidlin C.J., Dodson M.B., Madhavan L., Zhang D.D. (2019). Redox regulation by NRF2 in aging and disease. Free Radic. Biol. Med..

[220] Koháryová M., Kollárová M. (2015). Thioredoxin system—A novel therapeutic target. Gen. Physiol. Biophys..

[221] Lu J., Holmgren A. (2014). The thioredoxin antioxidant system. Free Radic. Biol. Med..

[222] Jones S.W., Luk K.C. (1988). Isolation of a chicken thioredoxin cDNA clone. Thioredoxin mRNA is differentially expressed in normal and Rous sarcoma virus-transformed chicken embryo fibroblasts. J. Biol. Chem..

[223] Tanaka Y., Tran P.O., Harmon J., Robertson R.P. (2002). A role for glutathione peroxidase in protecting pancreatic beta cells against oxidative stress in a model of glucose toxicity. Proc. Nat. Acad. Sci. USA.

[224] Wang D., Masutani H., Oka S., Tanaka T., Yamaguchi-Iwai Y., Nakamura H., Yang K.T., Lin C.Y., Huang H.L., Liou J.S. (2006). Control of mitochondrial outer membrane permeabilization and Bcl-xL levels by thioredoxin 2 in DT40 cells. J. Biol. Chem..

[225] Xiao R., Power R.F., Mallonee D., Routt K., Spangler L., Pescatore A.J., Cantor A.H., Ao T., Pierce J.L., Dawson K.A. (2008). Expressed transcripts associated with high rates of egg production in chicken ovarian follicles. Mol. Cell. Probes..

[226] Xiao R., Power R.F., Mallonee D., Routt K., Spangler L., Pescatore A.J., Cantor A.H., Ao T., Pierce J.L., Dawson K.A. (2012). Effects of yeast cell wall-derived mannan-oligosaccharides on jejunal gene expression in young broiler chickens. Poult. Sci..

[227] Marzoni M., Castillo A., Sagona S., Citti L., Rocchiccioli S., Romboli I., Felicioli A. (2013). A proteomic approach to identify seminal plasma proteins in roosters (Gallus gallus domesticus). Anim. Reprod. Sci..

[228] Hu L., Yu W., Li Y., Li Y., Guo J., Tang Z. (2015). Prokaryotic expression and antioxidant properties of mitochondrial thioredoxin-2 from broiler chicken. Chin. Vet. Sci..

[229] Yang J., Gong Y., Liu Q., Cai J., Zhang B., Zhang Z. (2018). Thioredoxin silencing-induced cardiac supercontraction occurs through endoplasmic reticulum stress and calcium overload in chicken. Metallomics.

[230] Smith A.D., Morris V.C., Levander O.A. (2001). Rapid determination of glutathione peroxidase and thioredoxin reductase activities using a 96-well microplate format: Comparison to standard cuvette-based assays. Int. J. Vitam. Nutr. Res..

[231] Gowdy K.M., Edens F.W., Mahmoud K.Z. (2015). Comparative Effects of Various Forms of Selenium on Thioredoxin Reductase Activity in Broiler Chickens. Int. J. Poult. Sci..

[232] Placha I., Takacova J., Ryzner M., Cobanova K., Laukova A., Strompfova V., Venglovska K., Faix S. (2014). Effect of thyme essential oil and selenium on intestine integrity and antioxidant status of broilers. Br. Poult. Sci..

[233] Lin S.L., Wang C.W., Tan S.R., Liang Y., Yao H.D., Zhang Z.W., Xu S.W. (2014). Selenium deficiency inhibits the conversion of thyroidal thyroxine (T4) to triiodothyronine (T3) in chicken thyroids. Biol. Trace Elem. Res..

[234] Zhao X., Yao H., Fan R., Zhang Z., Xu S. (2014). Selenium deficiency influences nitric oxide and selenoproteins in pancreas of chickens. Biol. Trace Elem. Res..

[235] Liang Y., Lin S.L., Wang C.W., Yao H.D., Zhang Z.W., Xu S.W. (2014). Effect of selenium on selenoprotein expression in the adipose tissue of chickens. Biol. Trace Elem. Res..

[236] Xu J.X., Zhang C., Cao C.Y., Zhu S.Y., Li H., Sun Y.C., Li J.L. (2016). Dietary Selenium Status Regulates the Transcriptions of Selenoproteome and Activities of Selenoenzymes in Chicken Kidney at Low or Super-nutritional Levels. Biol. Trace Elem. Res..

[237] Wang J., Liu Z., He X., Lian S., Liang J., Yu D., Sun D., Wu R. (2018). Selenium deficiency induces duodenal villi cell apoptosis via an oxidative stress-induced mitochondrial apoptosis pathway and an inflammatory signaling-induced death receptor pathway. Metallomics.

[238] Wang Y.X., Xiao X., Zhan X.A. (2018). Antagonistic effects of different selenium sources on growth inhibition, oxidative damage, and apoptosis induced by fluorine in broilers. Poult. Sci..

[239] Zhu Y., Jiao X., An Y., Li S., Teng X. (2017). Selenium against lead-induced apoptosis in chicken nervous tissues via mitochondrial pathway. Oncotarget.

[240] Zhang J., Bai K.W., He J., Niu Y., Lu Y., Zhang L., Wang T. (2018). Curcumin attenuates hepatic mitochondrial dysfunction through the maintenance of thiol pool, inhibition of mtDNA damage, and stimulation of the mitochondrial thioredoxin system in heat-stressed broilers. J. Anim. Sci..

[241] Han J.Y., Song K.D., Shin J.H., Han B.K., Park T.S., Park H.J., Kim J.K., Lillehoj H.S., Lim J.M., Kim H. (2005). Identification and characterization of the peroxiredoxin gene family in chickens. Poult. Sci..

[242] Lavric M., Maughan M.N., Bliss T.W., Dohms J.E., Bencina D., Keeler C.L., Narat M. (2008). Gene expression modulation in chicken macrophages exposed to Mycoplasma synoviae or Escherichia coli. Vet. Microbiol..

[243] Cao Z., Han Z., Shao Y., Geng H., Kong X., Liu S. (2011). Proteomic analysis of chicken embryonic trachea and kidney tissues after infection in ovo by avian infectious bronchitis coronavirus. Proteome Sci..

[244] Huang J., Ruan J., Tang X., Zhang W., Ma H., Zou S. (2011). Comparative proteomics and phosphoproteomics analyses of DHEA-induced on hepatic lipid metabolism in broiler chickens. Steroids.

[245] Lee S.H., Lillehoj H.S., Jang S.I., Jeong M., Kim D.K., Xu S., Lee S.K., Kim J.B., Park H.J., Kim H.R. (2014). Immune and anti-oxidant effects of in ovo selenium proteinate on post-hatch experimental avian necrotic enteritis. Vet. Parasitol..

[246] Cheng C.Y., Tu W.L., Chen C.J., Chan H.L., Chen C.F., Chen H.H., Tang P.C., Lee Y.P., Chen S.E., Huang S.Y. (2018). Functional genomics study of acute heat stress response in the smallyellow follicles of layer-type chickens. Sci. Rep..

[247] Drummond H.A., Mitchell Z.L., Abraham N.G., Stec D.E. (2019). Targeting Heme Oxygenase-1 in Cardiovascular and Kidney Disease. Antioxidants.

[248] Lever J.M., Boddu R., George J.F., Agarwal A. (2016). Heme Oxygenase-1 in Kidney Health and Disease. Antioxid. Redox. Signal..

[249] Kalinina E.V., Chernov N.N., Novichkova M.D. (2014). Role of glutathione, glutathione transferase, and glutaredoxin in regulation of redox-dependent processes. Biochemistry.

[250] Kasai S., Mimura J., Ozaki T., Itoh K. (2018). Emerging Regulatory Role of Nrf2 in Iron, Heme, and Hemoglobin Metabolism in Physiology and Disease. Front. Vet. Sci..

[251] Zhou S., Sun W., Zhang Z., Zheng Y. (2014). The role of Nrf2-mediated pathway in cardiac remodelling and heart failure. Oxid. Med. Cell Longev..

[252] Hayes J.D., Dinkova-Kostova A.T. (2014). The Nrf2 regulatory network provides an interface between redox and intermediary metabolism. Trends Biochem. Sci..

[253] Dayalan Naidu S., Kostov R.V., Dinkova-Kostova A.T. (2015). Transcription factors Hsf1 and Nrf2 engage in crosstalk for cytoprotection. Trends Pharmacol. Sci..

[254] Itoh K., Ye P., Matsumiya T., Tanji K., Ozaki T. (2015). Emerging functional cross-talk between the Keap1-Nrf2 system and mitochondria. J. Clin. Biochem. Nutr..

[255] Sihvola V., Levonen A.L. (2017). Keap1 as the redox sensor of the antioxidant response. Arch. Biochem. Biophys..

[256] Sahin K., Orhan C., Tuzcu M., Ali S., Sahin N., Hayirli A. (2010). Epigallocatechin-3-gallate prevents lipid peroxidation and enhances antioxidant defense system via modulating hepatic nuclear transcription factors in heat-stressed quails. Poult. Sci..

[257] Sahin K., Orhan C., Tuzcu M., Sahin N., Hayirli A., Bilgili S., Kucuk O. (2016). Lycopene activates antioxidant enzymes and nuclear transcription factor systems in heat-stressed broilers. Poult. Sci..

[258] Sahin N., Hayirli A., Orhan C., Tuzcu M., Akdemir F., Komorowski J.R., Sahin K. (2017). Effects of the supplemental chromium form on performance and oxidative stress in broilers exposed to heat stress. Poult. Sci..

[259] Zhang J.F., Bai K.W., Su W.P., Wang A.A., Zhang L.L., Huang K.H., Wang T. (2018). Curcumin attenuates heat-stress-induced oxidant damage by simultaneous activation of GSH-related antioxidant enzymes and Nrf2-mediated phase II detoxifying enzyme systems in broiler chickens. Poult. Sci..

[260] Zhang C., Chen K., Zhao X., Geng Z. (2018). Protective effects of resveratrol against high ambient temperature-induced spleen dysplasia in broilers through modulating splenic redox status and apoptosis. J. Sci. Food Agric..

[261] Lu Z., He X., Ma B., Zhang L., Li J., Jiang Y., Zhou G., Gao F. (2019). Dietary taurine supplementation improves breast meat quality in chronic heat-stressed broilers via activating the Nrf2 pathway and protecting mitochondria from oxidative attack. J. Sci. Food Agric..

[262] Habashy W.S., Milfort M.C., Rekaya R., Aggrey S.E. (2018). Expression of genes that encode cellular oxidant/antioxidant systems are affected by heat stress. Mol. Biol. Rep..

[263] Monson M.S., Cardona C.J., Coulombe R.A., Reed K.M. (2016). Hepatic Transcriptome Responses of Domesticated and Wild Turkey Embryos to Aflatoxin B_1_. Toxins.

[264] Liu Y., Wang W. (2016). Aflatoxin B1 impairs mitochondrial functions, activates ROS generation, induces apoptosis and involves Nrf2 signal pathway in primary broiler hepatocytes. Anim. Sci. J..

[265] Wang W.J., Xu Z.L., Yu C., Xu X.H. (2017). Effects of aflatoxin B1 on mitochondrial respiration, ROS generation and apoptosis in broiler cardiomyocytes. Anim. Sci. J..

[266] Wang H., Muhammad I., Li W., Sun X., Cheng P., Zhang X. (2018). Sensitivity of Arbor Acres broilers and chemoprevention of aflatoxin B(1)-induced liver injury by curcumin, a natural potent inducer of phase-II enzymes and Nrf2. Environ. Toxicol. Pharmacol..

[267] Muhammad I., Wang X., Li S., Li R., Zhang X. (2018). Curcumin confers hepatoprotection against AFB(1)-induced toxicity via activating autophagy and ameliorating inflammation involving Nrf2/HO-1 signaling pathway. Mol. Biol. Rep..

[268] Li S., Muhammad I., Yu H., Sun X., Zhang X. (2019). Detection of Aflatoxin adducts as potential markers and the role of curcumin in alleviating AFB1-induced liver damage in chickens. Ecotoxicol. Env. Saf..

[269] Chaudhary M., Rao P.V. (2010). Brain oxidative stress after dermal and subcutaneous exposure of T-2 toxin in mice. Food Chem. Toxicol..

[270] Yu M., Chen L., Peng Z., Wang D., Song Y., Wang H., Yao P., Yan H., Nüssler A.K., Liu L. (2017). Embryotoxicity Caused by DON-Induced Oxidative Stress Mediated by Nrf2/HO-1 Pathway. Toxins.

[271] Zhang C., Lin J., Ge J., Wang L.L., Li N., Sun X.T., Cao H.B., Li J.L. (2017). Selenium triggers Nrf2-mediated protection against cadmium-induced chicken hepatocyte autophagy and apoptosis. Toxicol. Vitr..

[272] Chen M., Li X., Fan R., Cao C., Yao H., Xu S. (2017). Selenium antagonizes cadmium-induced apoptosis in chicken spleen but not involving Nrf2-regulated antioxidant response. Ecotoxicol. Env. Saf..

[273] Wang J., Huang X., Zhang K., Mao X., Ding X., Zeng Q., Bai S., Xuan Y., Peng H. (2017). Vanadate oxidative and apoptotic effects are mediated by the MAPK-Nrf2 pathway in layer oviduct magnum epithelial cells. Metallomics.

[274] Ma Y., Zhu M., Miao L., Zhang X., Dong X., Zou X. (2018). Mercuric Chloride Induced Ovarian Oxidative Stress by Suppressing Nrf2-Keap1 Signal Pathway and its Downstream Genes in Laying Hens. Biol. Trace Elem. Res..

[275] Ma Y., Zheng Y.X., Dong X.Y., Zou X.T. (2018). Effect of mercury chloride on oxidative stress and nuclear factor erythroid 2-related factor 2 signalling molecule in liver and kidney of laying hens. J. Anim. Physiol. Anim. Nutr..

[276] Wang J., Yuan Z., Zhang K., Ding X., Bai S., Zeng Q., Peng H., Celi P. (2018). Epigallocatechin-3-gallate protected vanadium-induced eggshell depigmentation via P38MAPK-Nrf2/HO-1 signaling pathway in laying hens. Poult. Sci..

[277] Zheng X.C., Wu Q.J., Song Z.H., Zhang H., Zhang J.F., Zhang L.L., Zhang T.Y., Wang C., Wang T. (2016). Effects of Oridonin on growth performance and oxidative stress in broilers challenged with lipopolysaccharide. Poult. Sci..

[278] Zhang P., Zhong S., Wang G., Zhang S.Y., Chu C., Zeng S., Yan Y., Cheng X., Bao Y., Hocher B. (2018). N-Acetylcysteine Suppresses LPS-Induced Pathological Angiogenesis. Cell Physiol. Biochem..

[279] Ruan D., Fouad A.M., Fan Q.L., Chen W., Xia W.G., Wang S., Cui Y.Y., Wang Y., Yang L., Zheng C.T. (2018). Effects of corn dried distillers’ grains with solubles on performance, egg quality, yolk fatty acid composition and oxidative status in laying ducks. Poult. Sci..

[280] Gou Z.Y., Li L., Fan Q.L., Lin X.J., Jiang Z.Y., Zheng C.T., Ding F.Y., Jiang S.Q. (2018). Effects of oxidative stress induced by high dosage of dietary iron ingested on intestinal damage and caecal microbiota in Chinese Yellow broilers. J. Anim. Physiol. Anim. Nutr..

[281] Kang B., Wang X., Xu Q., Wu Y., Si X., Jiang D. (2018). Effect of 3-nitropropionic acid inducing oxidative stress and apoptosis of granulosa cells in geese. Biosci. Rep..

[282] Lu P., Xue W.Y., Zhang X.L., Wu D.W., Ding L.R., Wen C., Zhou Y.M. (2019). Heat-induced protein oxidation of soybean meal impairs growth performance and antioxidant status of broilers. Poult. Sci..

[283] Khaliq H., Wang J., Xiao L., Yang K.-L., Sun P.P., Lei C., Qiu W.-W., Lei Z., Liu H.-Z., Hui S. (2018). Boron Affects the Development of the Kidney Through Modulation of Apoptosis, Antioxidant Capacity, and Nrf2 Pathway in the African Ostrich Chicks. Biol. Trace Elem. Res..

[284] Ge J., Li H., Sun F., Li X.N., Lin J., Xia J., Zhang C., Li J.L. (2017). Transport stress-induced cerebrum oxidative stress is not mitigated by activating the Nrf2 antioxidant defense response in newly hatched chicks. J. Anim. Sci..

[285] Xu L., Zhang H.J., Yue H.Y., Wu S.G., Yang H.M., Qi G.H., Wang Z.Y. (2018). Low-current & high-frequency electrical stunning increased oxidative stress, lipid peroxidation, and gene transcription of the mitogen-activated protein kinase/nuclear factor-erythroid 2-related factor 2/antioxidant responsive element (MAPK/Nrf2/ARE) signaling pathway in breast muscle of broilers. Food Chem..

[286] Surai P.F. (2014). Polyphenol compounds in the chicken/animal diet: From the past to the future. J. Anim. Physiol. Anim. Nutr..

[287] Lee M.T., Lin W.C., Lee T.T. (2019). Potential crosstalk of oxidative stress and immune response in poultry through phytochemicals—A review. Asian-Australas. J. Anim. Sci..

[288] Lee M.T., Lin W.C., Wang S.Y., Lin L.J., Yu B., Lee T.T. (2018). Evaluation of potential antioxidant and anti-inflammatory effects of Antrodia cinnamomea powder and the underlying molecular mechanisms via Nrf2- and NF-κB-dominated pathways in broiler chickens. Poult. Sci..

[289] Lin X., Jiang S., Jiang Z., Zheng C., Gou Z. (2016). Effects of equol on H_2_O_2_-induced oxidative stress in primary chicken intestinal epithelial cells. Poult. Sci..

[290] Lin W.C., Lee M.T., Chang S.C., Chang Y.L., Shih C.H., Yu B., Lee T.T. (2017). Effects of mulberry leaves on production performance and the potential modulation of antioxidative status in laying hens. Poult. Sci..

[291] Niu Y., Zhang J.F., Wan X.L., Huang Q., He J.T., Zhang X.H., Zhao L.G., Zhang L.L., Wang T. (2019). Effect of fermented Ginkgo biloba leaves on nutrient utilisation, intestinal digestive function and antioxidant capacity in broilers. Br. Poult. Sci..

[292] Sahin K., Yenice E., Bilir B., Orhan C., Tuzcu M., Sahin N., Ozercan I.H., Kabil N., Ozpolat B., Kucuk O. (2019). Genistein Prevents Development of Spontaneous Ovarian Cancer and Inhibits Tumor Growth in Hen Model. Cancer Prev. Res..

[293] Ruan D., Zhu Y.W., Fouad A.M., Yan S.J., Chen W., Zhang Y.N., Xia W.G., Wang S., Jiang S.Q., Yang L. (2019). Dietary curcumin enhances intestinal antioxidant capacity in ducklings via altering gene expression of antioxidant and key detoxification enzymes. Poult. Sci..

[294] Jiang S.Q., Gou Z.Y., Lin X.J., Li L. (2018). Effects of dietary tryptophan levels on performance and biochemical variables of plasma and intestinal mucosa in yellow-feathered broiler breeders. J. Anim. Physiol. Anim. Nutr..

[295] Ruan D., Fouad A.M., Fan Q., Xia W., Wang S., Chen W., Lin C., Wang Y., Yang L., Zheng C. (2018). Effects of dietary methionine on productivity, reproductive performance, antioxidant capacity, ovalbumin and antioxidant-related gene expression in laying duck breeders. Br. J. Nutr..

[296] Bai W.K., Zhang F.J., He T.J., Su P.W., Ying X.Z., Zhang L.L., Wang T. (2016). Dietary Probiotic Bacillus subtilis Strain fmbj Increases Antioxidant Capacity and Oxidative Stability of Chicken Breast Meat during Storage. PLoS ONE.

[297] Bai K., Huang Q., Zhang J., He J., Zhang L., Wang T. (2017). Supplemental effects of probiotic Bacillus subtilis fmbJ on growth performance, antioxidant capacity, and meat quality of broiler chickens. Poult. Sci..

[298] Seidel U., Huebbe P., Rimbach G. (2018). Taurine: A Regulator of Cellular Redox Homeostasis and Skeletal Muscle Function. Mol. Nutr. Food Res..

[299] Kong B.W., Hudson N., Seo D., Lee S., Khatri B., Lassiter K., Cook D., Piekarski A., Dridi S., Anthony N. (2017). RNA sequencing for global gene expression associated with muscle growth in a single male modern broiler line compared to a foundational Barred Plymouth Rock chicken line. BMC Genom..

[300] Khatri B., Seo D., Shouse S., Pan J.H., Hudson N.J., Kim J.K., Bottje W., Kong B.C. (2018). MicroRNA profiling associated with muscle growth in modern broilers compared to an unselected chicken breed. Bmc Genom..

[301] Sivandzade F., Prasad S., Bhalerao A., Cucullo L. (2019). NRF2 and NF-κB interplay in cerebrovascular and neurodegenerative disorders: Molecular mechanisms and possible therapeutic approaches. Redox Biol..

[302] Moldogazieva N.T., Mokhosoev I.M., Feldman N.B., Lutsenko S.V. (2018). ROS and RNS signalling: Adaptive redox switches through oxidative/nitrosative protein modifications. Free Radic. Res..

[303] Stefanson A.L., Bakovic M. (2014). Dietary regulation of Keap1/Nrf2/ARE pathway: Focus on plant-derived compounds and trace minerals. Nutrients.

[304] Velichko O.A., Shabaldin S.V., Surai P.F. (2013). Practical aspects of vitagene concept use in poultry production. Poult. Poult. Prod. (Moscow).

[305] Shatskih E., Latipova E., Fisinin V., Denev S., Surai P. (2015). Molecular mechanisms and new strategies to fight stresses in egg-producing birds. Agric. Sci. Technol..

[306] Shatskih E., Latipova E., Nesvet E.G., Koburneev I.V. (2016). Usage of Antistress Preparations in Poultry Production.

